# Foot health education provision for people with rheumatoid arthritis–an online survey of UK podiatrists’ perceptions

**DOI:** 10.1186/s13047-016-0145-6

**Published:** 2016-04-26

**Authors:** Andrea S Graham, Anita E Williams

**Affiliations:** Centre for Health Sciences Research, University of Salford, Frederick Road, Salford, UK; Directorate of Prosthetics, Orthotics and Podiatry, University of Salford, Frederick Road, Salford, UK

**Keywords:** Patient education, Rheumatoid arthritis, Foot health, Podiatrist

## Abstract

**Background:**

Patient education supports general disease self-management and in relation to foot problems, it is recommended as a key intervention for people with rheumatoid arthritis (RA). Further, it is known what the foot health educational (FHE) needs are in relation to their experiences of foot problems. Podiatrists are the key health professionals who provide the management of RA-related foot pathology and this includes the delivery of FHE. However, we do not know what is currently provided and what podiatrists’ perceptions are of this intervention. It is possible that there is a difference between what is provided and what patients need in order to maximise their foot health benefits and hence this may contribute to the persistence of foot problems and symptoms. This study primarily aims to define what UK podiatrists’ perceptions of FHE are in relation to; what is delivered, how it is delivered, and the timing of its delivery, in the context of its’ accessibility. The secondary aim is to identify any influence of the participants’ gender, age and duration of professional qualification on their responses.

**Method:**

An online survey of UK HCPC registered podiatrists was used to capture quantitative data in relation to the perceived; aims, content, methods and effectiveness, timing and barriers to FHE provision to people with RA. Data was analysed to assess significant associations between the participant responses and their gender, age and duration of professional qualification. Free text comments were analysed using thematic analysis.

**Results:**

43 podiatrists across the UK completed the survey. The majority of participants stated that, they provided FHE and agreed with its overall aims. The most common methods of delivery that were perceived to be most effective were: verbal, written and website based information. The best times at which to deliver FHE were thought to be at the point of diagnosis of RA and at any available opportunity of health care delivery. The majority of participants thought they had enough knowledge and access to information resources to effectively deliver FHE, but half of the participants felt that consultation duration limited their ability to do so. Gender and duration of professional qualification influenced participants’ perceptions of FHE.

**Conclusion:**

The importance and content of FHE for people with RA has been defined, but time limitations are seen to restrict its delivery. The development of an education needs analysis tool to facilitate efficient identification of patients FHE needs could enable timely and tailored delivery of FHE to people with RA.

**Electronic supplementary material:**

The online version of this article (doi:10.1186/s13047-016-0145-6) contains supplementary material, which is available to authorized users.

## Background

Foot health education is recommended as a key intervention for people with rheumatoid arthritis (RA) related foot problems [1, 2] in order to support self-management. Podiatrists are ideally placed to provide foot health education (FHE) as an intervention [1]. As up to 80 % of people with RA will develop foot-related pathology throughout the duration of their disease [3, 4], even when the disease is in remission, there is clearly a need for foot health interventions [1] and the inclusion of FHE as in intervention in its own right.

We know that patient education that supports disease self-management is effective in improving patient knowledge [5, 6], self-efficacy [7], disease activity scores [5], functional ability [6], mental health status [7] and in reducing pain [7]. Hence it could be considered essential for podiatrists to provide specific patient education that could improve self-management of foot problems, which are a significant burden to those with RA.

There are no specific FHE interventions for people with RA [8] therefore in order to develop and evaluate the potential effectiveness of FHE as a definable intervention for people with RA, there is a need to understand what its possible key components are and how it works. In gaining an understanding of this, the development of FHE as an intervention will align with the modelling phase of the MRC Complex Intervention Framework [9].

We know from previous work what people with RA have experienced and what they need in relation to foot health education (FHE) [10]. However, given that podiatrists are the main providers of FHE, we need to know the methods, timing, content and effectiveness of its provision, together with the potential influences on the delivery of FHE. This knowledge is key in defining the information ‘needs’ of both the patient and practitioner. Foot health information that is tailored for the individual can potentially improve patient adherence to foot health interventions and therefore positive foot health outcomes in this patient group [11]. Further, exploratory work has indicated that people with RA [10] and podiatrists [12] perceive that factors such as gender, age and time since qualification (podiatrists) may also influence the provision of FHE in relation to the therapeutic relationship.

Therefore the primary aim of this study was to understand podiatrists’ opinions and perceptions about FHE for people with RA. The secondary aim was to identify the current status of RA-related FHE provision in the UK and what may influence this, for example; gender, age and duration of time since qualification. Podiatrists’ opinions on what should be delivered, how it should be delivered and at what point in the persons’ experience of foot problems it would be most effective, are not known. To date, this has not been explored and has the potential to contribute significantly in relation to the provision of foot health education, not just by podiatrists but by any professional involved in managing people with RA who have foot problems.

## Methods

The study was granted ethical approval from the University of Salford, Research Innovation and Academic Engagement Ethical Approval Panel (HSCR12/35).

### Survey questionnaire design

The survey questionnaire was designed to capture quantitative data from podiatrists. Questions were developed from a literature search and the results of previous focus group work with UK National Health Service (NHS) podiatrists, which informed the content of the questionnaire [10, 12]. To ensure face and content validity the questionnaire was piloted with four UK NHS podiatrists that work within rheumatology. ‘Think aloud’ cognitive debriefing [13, 14] was used in order to reduce sources of response error, ensure clarity of questions and refine the overall structure of the questions. The results of the pilot led to a small number of changes to improve the clarity of the question completion instructions.

The final survey consisted of five sections, plus demographics (Additional file 1) with 17 questions in total.

1. Aims of Foot health education
2. The best ways of providing foot health education
3. What should be included in foot health education provision
4. When is the best time to provide foot health education
5. Accessing foot health education/information

A free text comment section was included for additional comment.

The questionnaires were anonymous, self-administered and of a cross-sectional observational design using a web based survey through the Bristol Online Survey website (https://www.onlinesurveys.ac.uk/). A mixture of open-ended, closed-ended dichotomous, contingency, nominal and ordinal polytomous questions were used to reduce the risk of missing data [15, 16].

### Participants

Inclusion criteria were: podiatrists with current Health and Care Professions Council (HCPC) registration, working within the UK National Health Service and with access to the Internet. The participants were recruited between September and November 2013, through the Podiatry JISC-Mail service, via e-mail invitation with a web-link to the survey. A second ‘reminder’ e-mail was sent after 2 weeks. Consent was implicit by the completion of the survey and participants were informed of this at the start of the survey.

### Data analysis

Data was analysed using SPSS v 20.0 (SPSS, Chicago, IL, USA). The primary analysis was descriptive statistics. Secondary analyses were cross-tabulation; Fishers Exact test was performed to determine the strength of any associations between the participants’ demographic variables of Gender, Age Range, Years Qualified and the responses to the items in section 2–6. Fishers Exact test was applied where cell frequencies in 2x2 cross-tabulated contingency tables was less than 5. A *p* < 0.05 was considered to indicate statistical significance (Additional file 2).

Free text comments (Additional file 3) were subject to thematic analysis by the primary author (AG) to develop a thematic framework using the six-step approach outlined by Braun and Clarke [17] and to illustrate the main themes within the comments provided. The thematic framework was agreed by the co-author (AW) to evaluate validity of the data [18].

## Results

### Demographics

42 podiatrists (f = 31, m = 11) completed the survey (Table 1), all were Health and Care Professions Council registered.

**Table 1:** Participant Demographics

Gender		Female (n)	Male (n)	Total
(S.D = 0.45)		31	11	42
Age Range (S.D = 0.89)	21-30 years	2	0	2
	31-40 years	10	4	14
	41-50 years	12	5	17
	51-60 years	7	1	8
	More than 60 years	0	1	1
Duration of time qualified	up to 1 year	1	0	1
	2-5 years	1	1	2
	5-10 years	4	2	6
	10-20 years	14	2	16
	20-30 years	9	4	13
	30-40 years	2	2	4
HCPC registered		31	11	42
Service type	Primary Care	15	8	23
	Secondary Care	13	2	15
	Equal Split	3	1	4
Geographic location	SE England	3	0	3
	NW England	17	3	20
	SW England	2	2	4
	Greater London	0	0	0
	West Midlands	1	0	1
	East Anglia	0	0	0
	Yorkshire/N Humberside	2	0	2
	East Midlands	3	0	3
	S Central England	2	0	2
	NE England	0	2	2
	Wales	0	0	0
	Scotland	1	3	4
	N. Ireland	1	0	1

### Results from the survey

#### Aims of foot health education

**Fig. 1:**
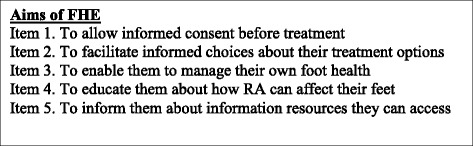
Section 2 survey items: the aims of foot health education. Legend: Fig. 1 shows the items that constitute section 2 of the FHE survey in relation to the AIMS of FHE

The majority of podiatrists (88 %, *n =* 37) agreed with the aims of foot health education (Fig. 1). Two podiatrists disagreed with item 1.

All items, were statistically significant (*p* = <0.05) in relation to duration of years qualified and the gender of the participants. Participants who had been qualified for over 10 years and female tended to agree more strongly with the aims of FHE. Only one item, ‘To inform patients about information resources they can access’ did not reach statistical significance.

#### The best ways of providing/receiving foot health education

97.6 % (*n =* 40) stated that they provided FHE. The methods of delivery were, verbal information (97.5 %, *n =* 39), written information (69 %, *n =* 29) and signposting patient to websites (57.5 %), *n =* 24). The relationship between the provision of verbal foot health information and the gender of the participants approached statistical significance (*p* = 0.064), with 100 % (*n =* 31) female participants stating that they provided verbal foot health information in comparison to 82 % (*n =* 9) of males. There were no other statistically significant results in relation to methods of FHE delivery.

Other methods of delivery such as group education sessions and the use of audio-visual aids such as DVDs, self-care demonstrations or the specific uses of images to aid delivery of education are infrequently used.

In relation to the effectiveness of the methods of delivery, written (76 %, *n =* 32) and verbal (100 %, *n =* 42) provision were ranked the highest, followed by website based information (62.8 %) [Arthritis Research UK (ARUK), *n =* 22; Arthritis Care *n =* 16; National Rheumatoid Arthritis Society (NRAS) *n =* 15].

There was no statistically significant relationship between the age, gender or years qualified and perceived effectiveness of any method of FHE with the exception of verbal information which approached statistical significance for gender (*p* = 0.069), with females tending to rate verbal information as more effective than men and years since qualification (*p* = 0.081), with participants who have been qualified longer (>20 years) finding verbal information to be less effective than those with fewer years since qualifying.

#### The content of foot health education

All of the participants considered all the items to be important or very important with gender being the only independent variable to have a statistically significant relationship (*p* = <0.05) in relation to the following items: signs and symptoms of foot problems related to RA, management options relating to foot health and how patients should manage their own foot health. Female participants attributed a higher level of importance to these items of FHE content, than male participants.

#### The timing of foot health education

**Fig. 2:**
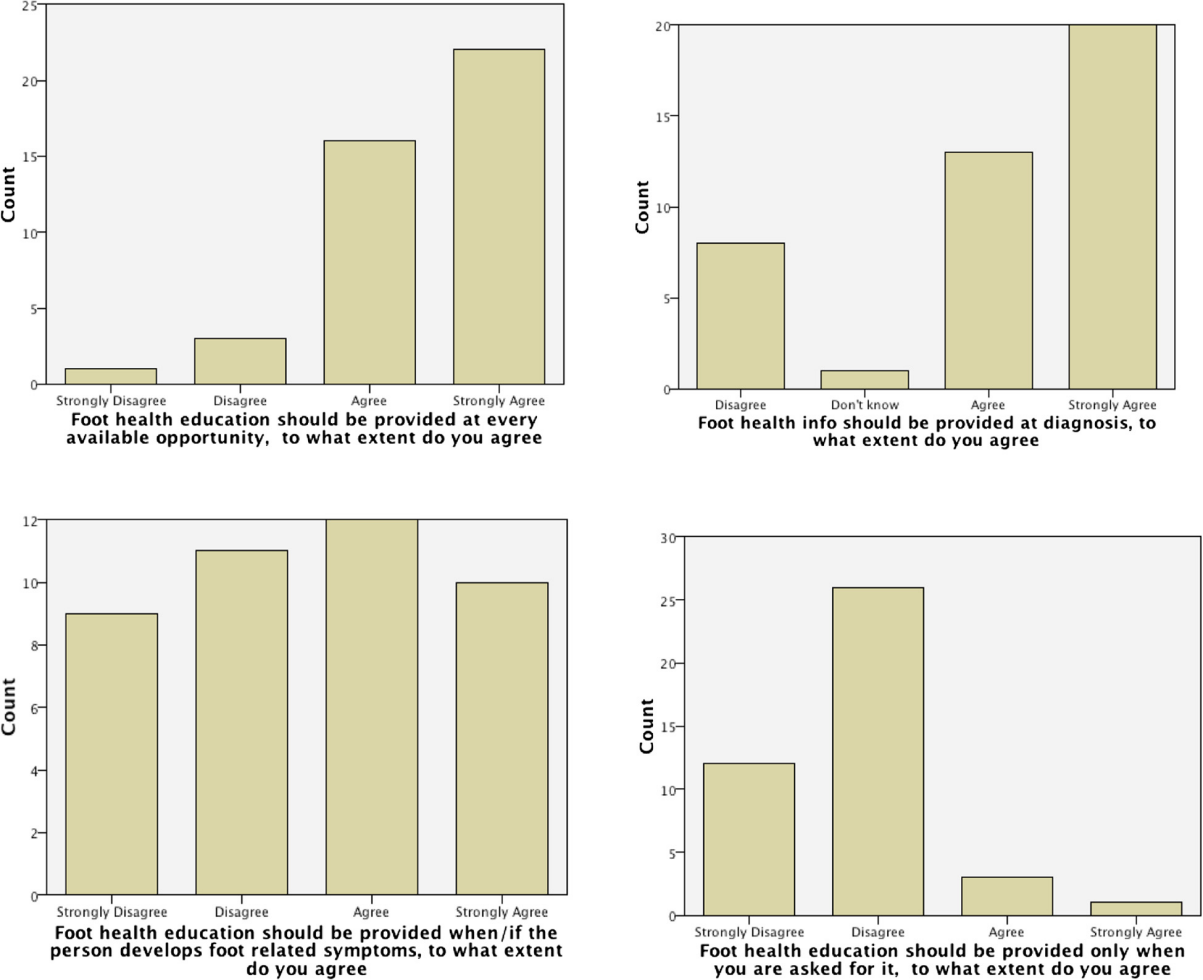
Agreement with the timing of FHE. Legend: Bar charts show the level to which podiatrists’ agree with items for the timing of FHE provision

78.6 % (*n =* 33) of participants agree that patients should be provided with FHE at the point of diagnosis and 90.5 % (*n =* 38) think it should be provided at every available opportunity but disagree that FHE should only be provided when asked for it by the patient. However, the participant’s opinion was split equally when asked about providing FHE when the patient develops foot related symptoms; 47.6 % (*n =* 20) disagreed whilst 52.4 % (*n =* 22) agreed (Fig. 2).

There was a statistically significant relationship between the years since qualification and the items: ‘FHE should be provided only when asked for it’ (*p* = 0.034), participants who had been qualified more than 30 years were more likely to disagree with this statement and ‘FHE should be provided when or if the person develops foot-related symptoms’ (*p* = 0.022). Participants that had been qualified for duration of time of more than 5 years were more likely to agree with this statement.

#### Accessing and barriers to the provision of foot health education/information

54.8 % (*n =* 23) participants thought there was enough time during consultations to provide FHE. The majority (78 %, *n =* 33) of participants stated that they had access to RA-specific foot health information such as leaflets and that the patients they treated used it. The majority of participants (92.9 %, *n =* 39) stated that they had enough knowledge about how RA affected the feet in order to provide effective FHE. However, approximately 30 % (*n =* 13) stated that patients did not use the FHE provided due to financial constraints or that it lacked personal relevance.

The only item to reach statistical significance was ‘You have access to foot health information’ in relation to the gender of participants (*p =* 0.031), with more female participants strongly agreeing with the statement compared with males who either agreed or strongly disagreed. There was no statistically significant relationship between the genders, the age or the duration of years qualified and perceived barriers to FHE provision.

#### Thematic analysis of free text comments

There were seven questions that allowed free text comments within the survey. 14 free text comments were provided in total for sub-questions 15 and 11 for sub-questions 16. Eleven participants provided additional free text comments within question 17, the ‘Any other comments’ section (Table 2).

**Table 2:** Outline of the basic and organising themes developed from the thematic analysis

Basic Themes	Organising Themes
Time restriction in consultations	Influence of time
Timing of delivery–	
Limited financial resources	Limited Resources
Limited knowledge of impact of RA on feet	
Limited access to group education sessions or patient support group sessions	
Gender influence on engagement with footwear advice	Footwear and behaviour change
Influence of Age/occupation of patient on engagement with footwear advice	
Influence of patients negative perceptions of podiatrist-advised footwear styles	
Too soon–overwhelming/lacks relevance	Negative impact of information provision
Too late–damage already done	
Can be perceived as ‘threatening’ if provided ‘incorrectly’	

## Discussion

This study has been the first to describe the opinions and perceptions of NHS podiatrists about RA related FHE in relation to its’ aims, method and timing of delivery, its’ content and potential barriers to its provision. Given the re-profiling of many NHS specialist podiatry services, resulting in reduced access to podiatrists, it is crucial that FHE is provided in a way that supports self-efficacy and self-management by all healthcare practitioners that are involved in the management of people with RA. This work will inform practitioners from a specialist and professional context, what patients need in relation to self-care, so that those people who do develop serious foot problems can be seen by the few specialists that remain and also prevent problems from having a more significant impact upon the individual.

The response rate for this study represents 50 % of the sample population invited to participate, which is deemed an acceptable rate for a survey method of data collection. Responses came from participants working in both UK Primary Care (health care services directly accessed by patients) and UK Secondary Care (health care services that generally require General Practitioner referral), although a question about their experience within the specialist area of Rheumatology was not included and may have provided insight about how their experience influenced their responses. Responder bias should remain a consideration in the interpretation of the results as it is possible that the respondents were those that had an interest in the subject area and we cannot know if the responses of those who did not complete the survey would have been different [19]. In addition, although there was a geographical spread of participants across the UK, the majority were based in the North West of England and therefore the secondary aim of the study was not fully achieved. The primary aim of the study was achieved by providing insight about how FHE for people with RA is perceived by podiatrists, the barriers and influences upon its provision.

The majority of participants agreed with the aims of FHE and stated that they provided some FHE to people with RA as part of their overall foot care. However, many people with RA are unable or unaware that they can access NHS podiatry services and thus are denied access to podiatrists who are considered a key information resource [20]. Further to this, for some people who do receive podiatry care, they perceive that podiatrists and other health care practitioners lack knowledge of how RA can impact on both the foot and the individual [21]. Hence if health care practitioners are perceived to lack insight into the bio-psychosocial impact of RA on foot health, then they may not be able to provide the FHE that patients need. This may be reflective of a training need across the health care professions that are involved in the management of people with RA, not just podiatrists.

In this study the majority of the participants felt that they had enough knowledge to allow them to provide effective FHE to people with RA. Indeed, females were more likely to access information resources to support FHE, aligning with the work of Roter et al.,[22] who found that female health care providers were more patient-centred and spent more time on psychosocial/socio-emotional exchange than males during the consultation. This poses a challenge in relation to recommendations. However, it may be that female gender traits lend more to this supportive action and this approach could be part of under and post-graduate training. In this study, thematic analyses of the free text data identified podiatrists’ perceptions that; the patients’ gender, age and historical perceptions of footwear for example, potentially influenced their engagement with positive foot health behaviours. This is echoed in the findings of research undertaken with people with RA, where the impact of having limited footwear as a female with RA has been poignantly expressed [21, 23]. Understanding the reasons why a person with RA may be ‘resistant’ to change in relation to foot health behaviour may assist practitioners in developing a more patient-centred approach to the provision of FHE.

Further, the years of post-qualification practice also appeared to influence the participant’s opinions and perceptions of FHE. The more novice podiatrists may not have the experience for managing the more complex patient needs in a time limited consultation [12] or have developed the insight to identify when patients are more likely to be receptive to the provision of FHE [24]. Identification of a persons readiness to engage in positive health behaviour change is a key component of a patient-centred approach to the consultation [24]. Firmly embedding the use of motivational interviewing techniques in the undergraduate curriculum, together with rigorous assessment and developmental feedback with respect to communication skills may help to equip undergraduate healthcare practitioners with the skills to manage complex patient needs and ensure similarities in communication skills development between male and female undergraduates.

Many identified the lack of time within the consultation and lack of resources as a barrier to being able to focus on anything other than the physical needs of the patient and this is consistent with the findings of previous work with both people with RA and podiatrists [10, 12, 21]. This lack of time reduces or removes the opportunity for a podiatrist to provide patient focussed FHE based on their physical, but also their psychological and social needs.

Despite the barriers of lack of time and inexperience, the participants did value FHE and identified what should be provided and tailored to their patients’ individual needs and priorities. In order to achieve this in a time limited consultation, podiatrists need to identify what the patients’ needs and priorities are. An Educational Needs Analysis Tools (ENAT) has been developed and validated for use in people with RA to facilitate timely and relevant patient education [25]. A specific foot health educational needs assessment tool may efficiently identify what the patient’s requirements are. However, until this tool is developed, we recommend that as a minimum, podiatrists should ask about what their patients would like to know and signpost them to the appropriate resources such as web sites or leaflets. Indeed, leaflets and other locally produced written information were reported to be the main vehicle for FHE. The use of combined methods of FHE delivery, such as verbal information being reinforced with written information, aligns with research findings that demonstrated that such an approach is the most effective in the provision of general RA information [26].

Over half of the participants stated that they do direct patients to RA or arthritis specific web sites such as Arthritis Research UK (www.arthritisresearchuk.org), Arthritis Care (www.arthritiscare.org.uk) and the National Rheumatoid Arthritis Society (www.nras.org.uk). These provide flexible, on-demand access to information and peer support [27]. In addition, patients can choose to access information that is the most pertinent to them at that point in time, thereby tailoring it to their own needs. Therefore, people with RA should be directed to the web-based resources if they are able to access the Internet and/or provided with foot health specific leaflets.

The participants viewed all content items for FHE as being either important or very important in agreement with the results from work with people with RA [10]. The fact that the participants place such high value upon all items in relation to the educational content, suggests that FHE needs to be considered as an intervention in itself. Further, considering ‘education provision’ as a treatment modality aligns with the need for healthcare practitioners being ethically obliged to provide patients with enough information about their disease and its management options in order to facilitate informed consent [28]. Therefore, it could be argued that ‘education provision’ should be viewed as a distinct entity from the provision of information which is an ethical ‘must’.

**Fig. 3:**
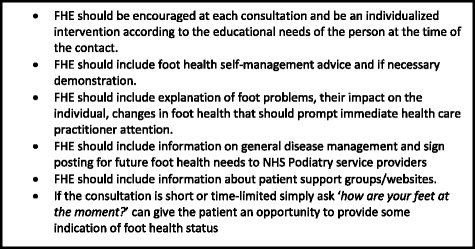
Components of FHE for people with RA. Legend: Fig. 3 highlights the key minimum FHE components that should be provided to people with RA

The timing of FHE was considered important and the participants considered that FHE should be provided at the point of diagnosis and at every available opportunity. Equally they agreed that they shouldn’t wait to provide information until patients asked for it. Despite the knowledge that many people can feel overwhelmed with too much information upon their initial diagnosis [29], there is a need to ensure that people have information at a point in time that allows them to self-manage from as early as possible [30]. It is recognised that foot and general health educational needs are temporal, in relation to the fluctuating nature of the disease and in relation to the individual’s ability to adjust to their diagnosis [12, 24]. Hence, providing people with RA an opportunity at each consultation to identify their educational needs, will allow them to ask questions that are pertinent to the current state of their feet and general health. Further to this it will enable the practitioner to contextualize their educational needs by attempting to understand the motivation that underlies the persons health behaviour goals. This ‘*person-in-context’* approach [31] enables the practitioner to identify the influence of the psychological, cognitive, self-efficacy beliefs, demographic, environmental and situational factors upon their information needs, as outlined by the Wilson Model [25]. Understanding such an approach should enable practitioners to fully consider; why, what and how to meet the FHE needs of patients in practice [32]. This study has identified what the components of FHE should be (Fig. 3) in relation to what people with RA need in order to reduce foot symptoms and maximise their foot health. Figure 3 outlines the general components of foot health education that podiatrists and other health professionals should aim to provide dependant upon the needs of the person with RA.

## Conclusion

In order to reduce the impact and burden of foot problems on people with RA, there needs to be a tailored and timely approach to FHE provision that both supports self-management and that takes into account the patients’ needs over the course of their disease journey. The podiatrists have defined the importance and content of FHE from a specialist professional perspective, but as a primary intervention delivered by them in a time limited consultation; it is relegated to an adjunct to treatment rather than an intervention in its own right.

Future research will be focussed on the development and validation of a simple foot health needs analysis tool so that patients can easily and accurately identify both their needs for foot health interventions (including specific FHE) and signposting for FHE that supports self-management.

## Additional files

Additional file 1: Practitioner Survey of FHE for people with RA. The file includes the survey questions and all raw data of the responses. (PDF 113 kb) Additional file 2: Table of results from statistical analyses. The table illustrates the influence of the years qualified, age range and gender of the podiatrists on their survey responses. (DOCX 187 kb) Additional file 3: Free text comments from RA FHE survey of podiatrists. This file shows the free text comments taken from Rheumatoid Arthritis foot health education survey for practitioners. (DOCX 24 kb)

## Abbreviations

ARUK: arthritis research UK
ENAT: educational needs analysis tool
FHE: foot health education
HCPC: health and care professions council
NHS: national health service
NRAS: national rheumatoid arthritis society
RA: rheumatoid arthritis
