# Supplementary material for: Foot health education provision for people with rheumatoid arthritis–an online survey of UK podiatrists’ perceptions
Source: J Foot Ankle Res. 2016 Apr 26;9:13. doi: 10.1186/s13047-016-0145-6 (PMC4845353; doi:10.1186/s13047-016-0145-6)
Supplement: Additional file 1: — Practitioner Survey of FHE for people with RA. The file includes the survey questions and all raw data of the responses. (PDF 113 kb) [file 13047_2016_145_MOESM1_ESM.pdf]

Rheumatoid Arthritis foot health education survey for practitioners

Showing 42 of 42 responses

Showing **all** responses

Showing **all** questions

Section 1

1 Are you

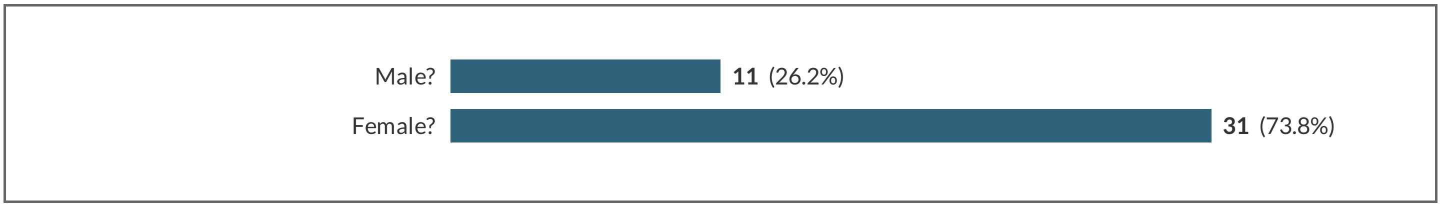

2 What age range are you? please select one option from the list.

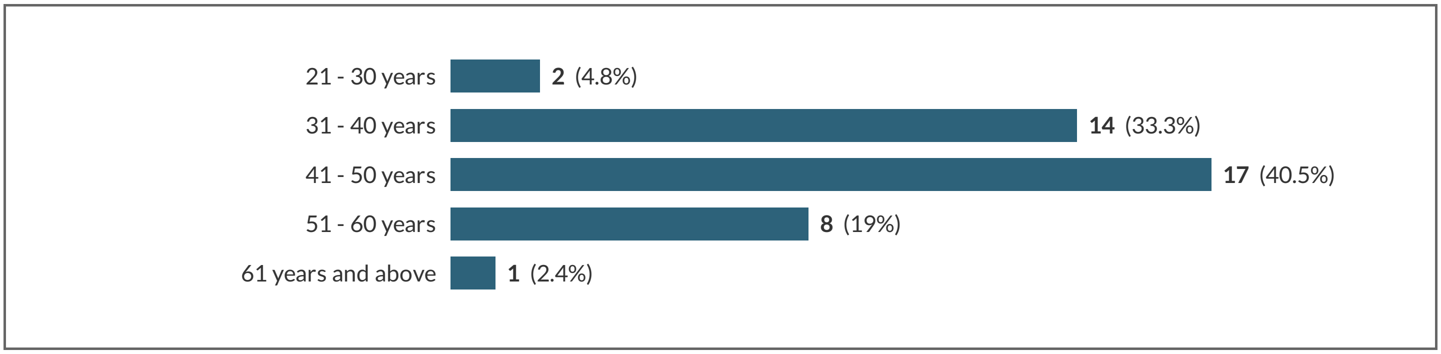

3 Please select the length of time since you qualified as a podiatrist.

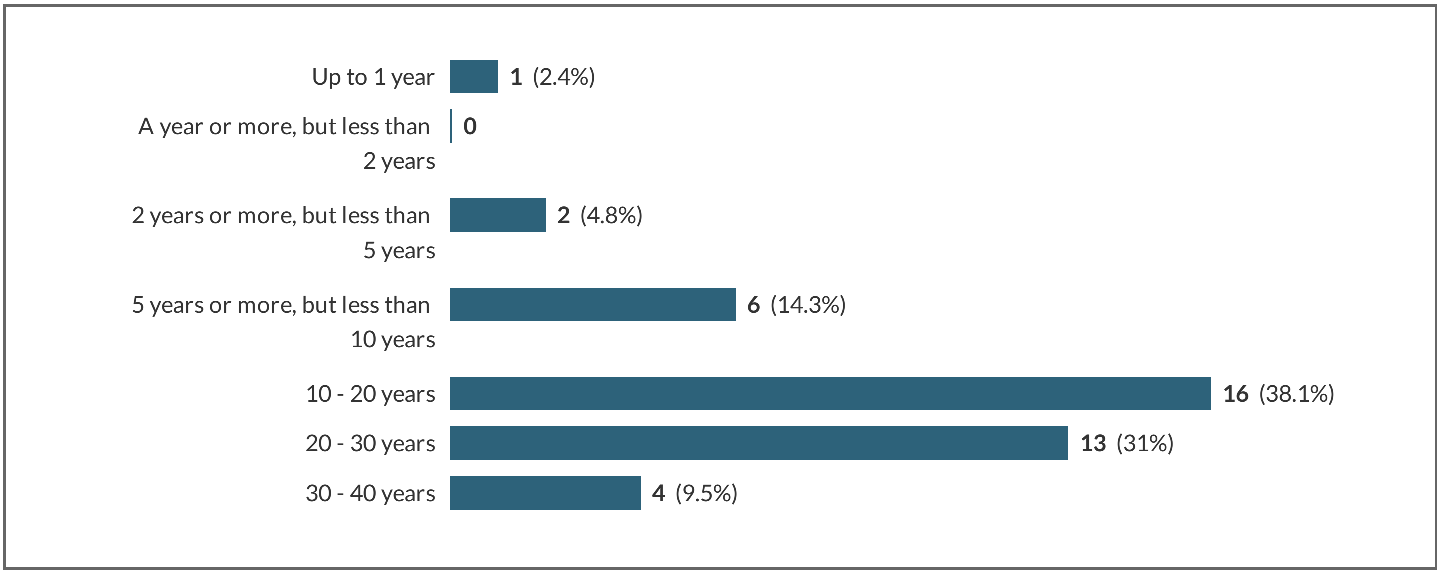

4 Are you registered with the Health & Care Professions Council?

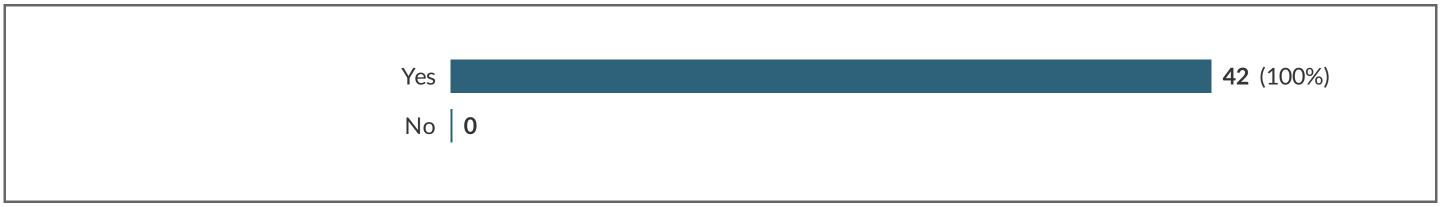

5 Please select where you practice for the MAJORITY of the time.

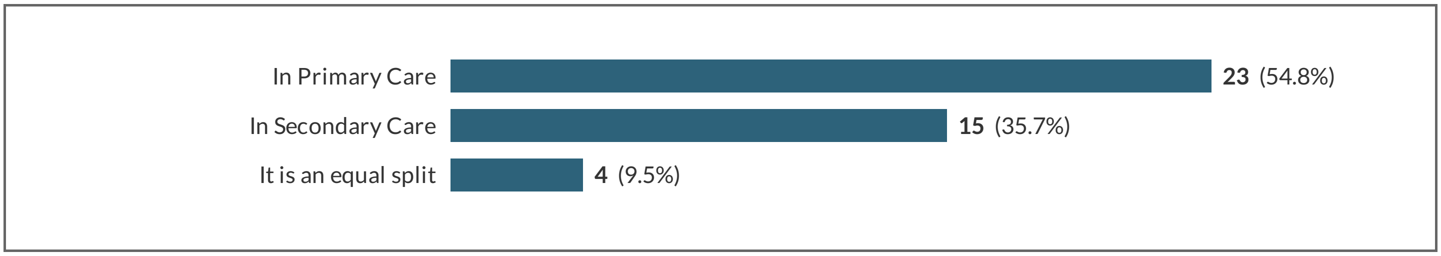

6 Do you work in the UK or elsewhere?

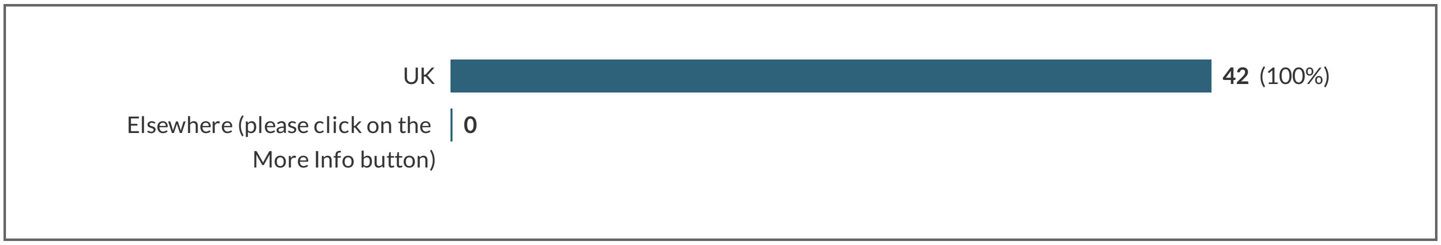

7 Which UK geographic location do you work within? (select ONE area only)

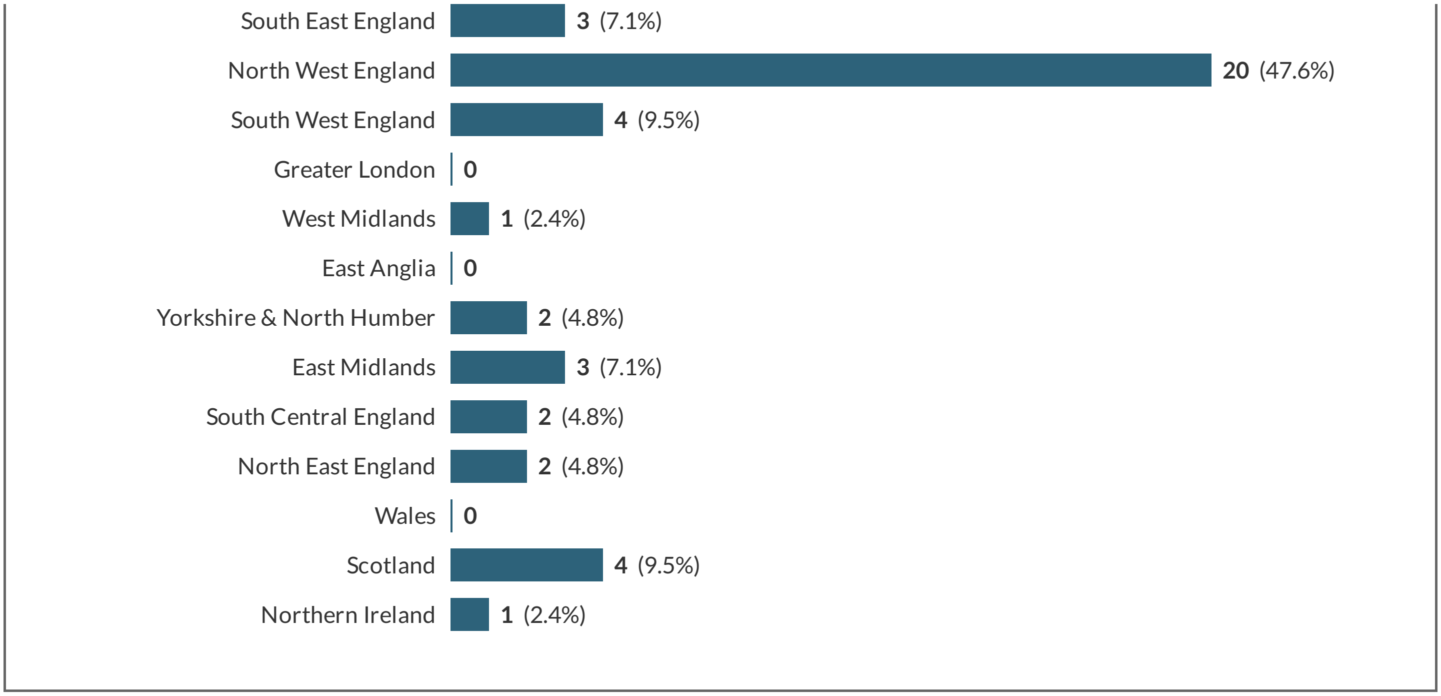

Section 2: The aims of foot health education.

8 To what extent do you agree with the following statements in relation to the AIMS of foot health education.

8.1 To allow informed consent before treatment

8.1.a To allow informed consent before treatment - Select the level to which you agree for each statement

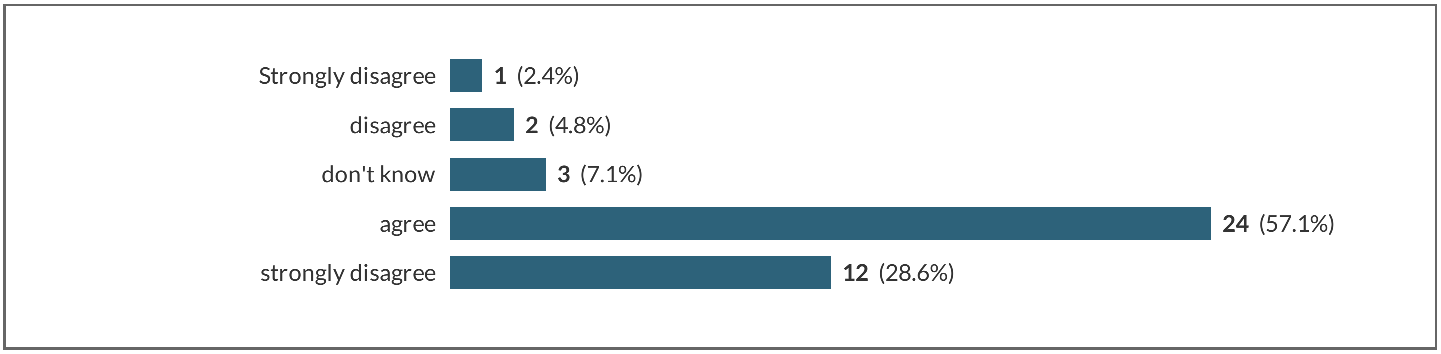

8.2 To facilitate informed choices about their treatment options

8.2.a To facilitate informed choices about their treatment options - Select the level to which you agree for each statement

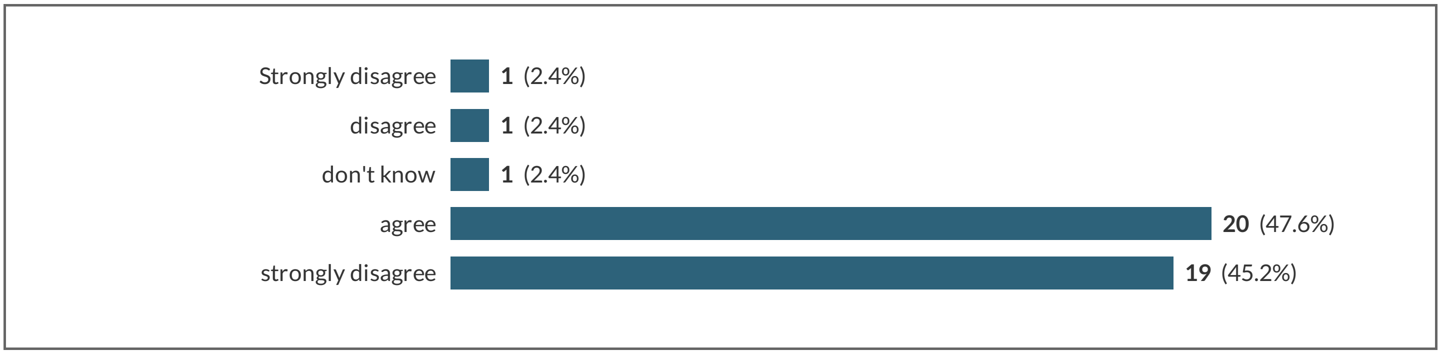

8.3 To enable them to manage their own foot health

8.3.a To enable them to manage their own foot health - Select the level to which you agree for each statement

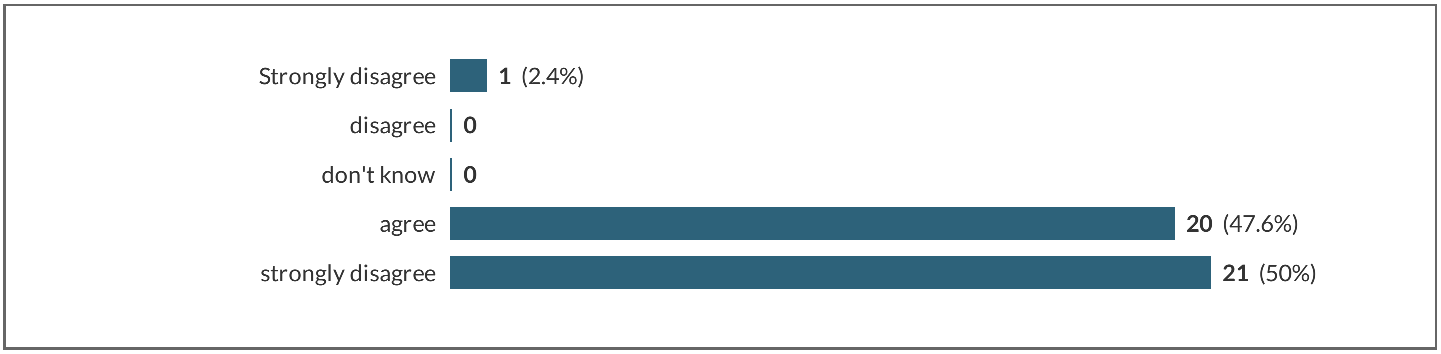

8.4 To educate them about how RA can affect their feet

8.4.a To educate them about how RA can affect their feet - Select the level to which you agree for each statement

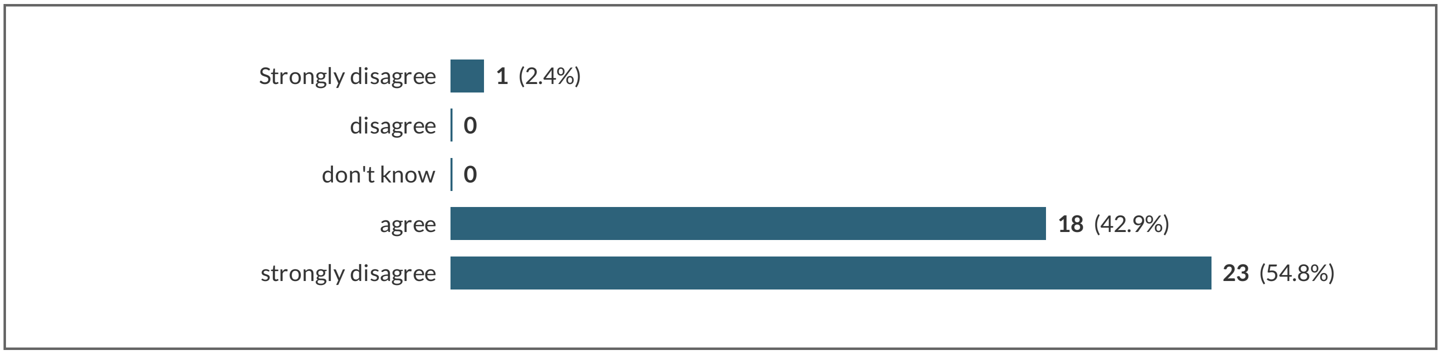

8.5 To inform them about information resources they can access

8.5.a To inform them about information resources they can access - Select the level to which you agree for each statement

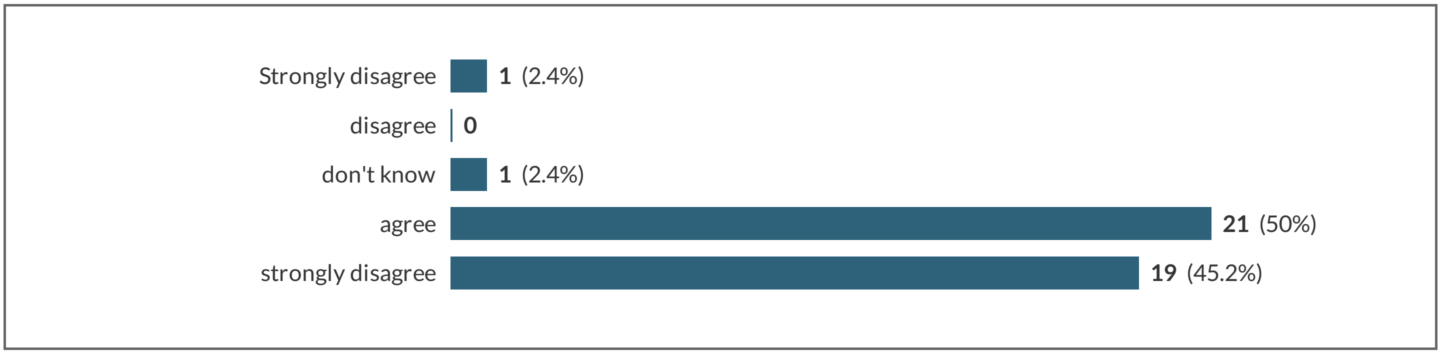

Section 3: Methods of providing foot health education

9 Do you provide people with RA, foot health information/ education?

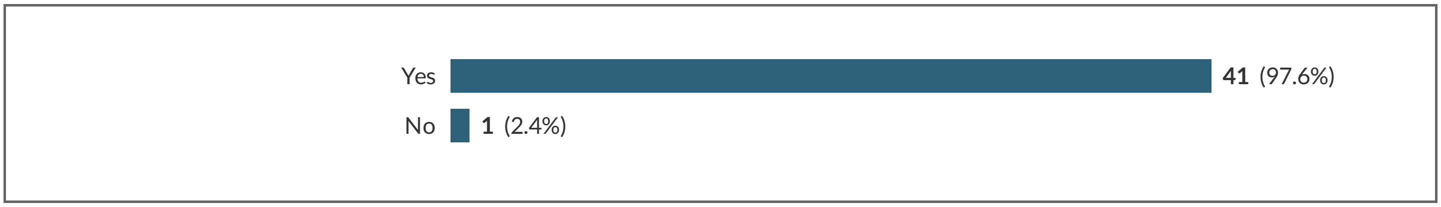

10 If you answered YES please indicate which types of information you have provided from the list below. Select all that apply.If NO, please move onto the next question.

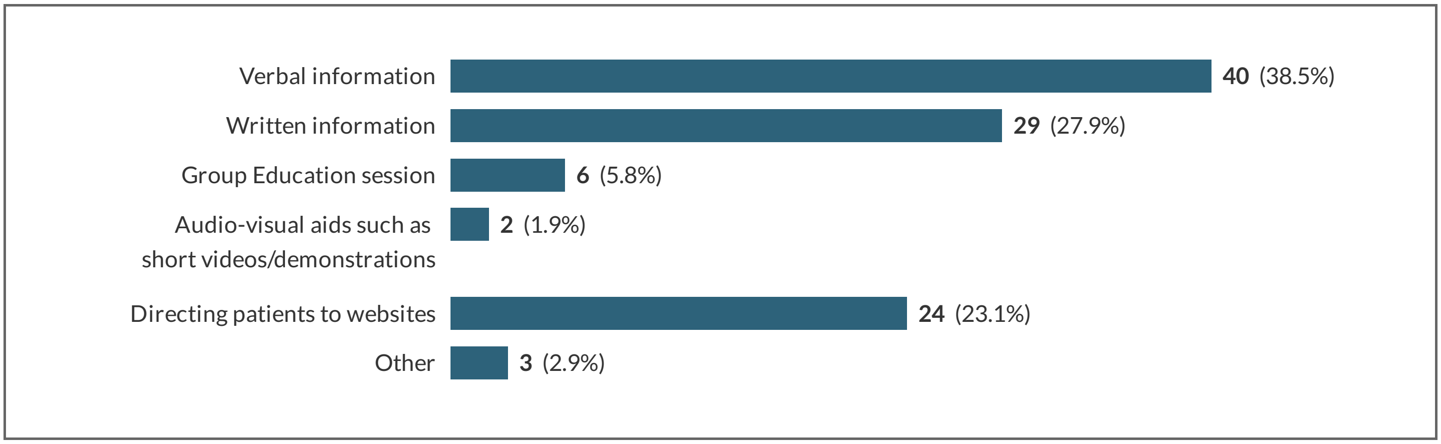

10.a If you selected Other, please specify:

**Showing all 3 responses**	
Weekly MDT clinic for newly diagnosed infl.arthritis shared with rheum. physio and rheum O.T. Used to hold group session but feedback better for this format	120812-120806-6760694
leaflets are available if needed	120812-120806-6760696
Examples of footwear in the clinic	120812-120806-6760704

11 If you direct your patients to a foot health or disease-related website can you please select from the list below the ALL ones that you use. If there are websites we have not included, please list them in the txet box available.

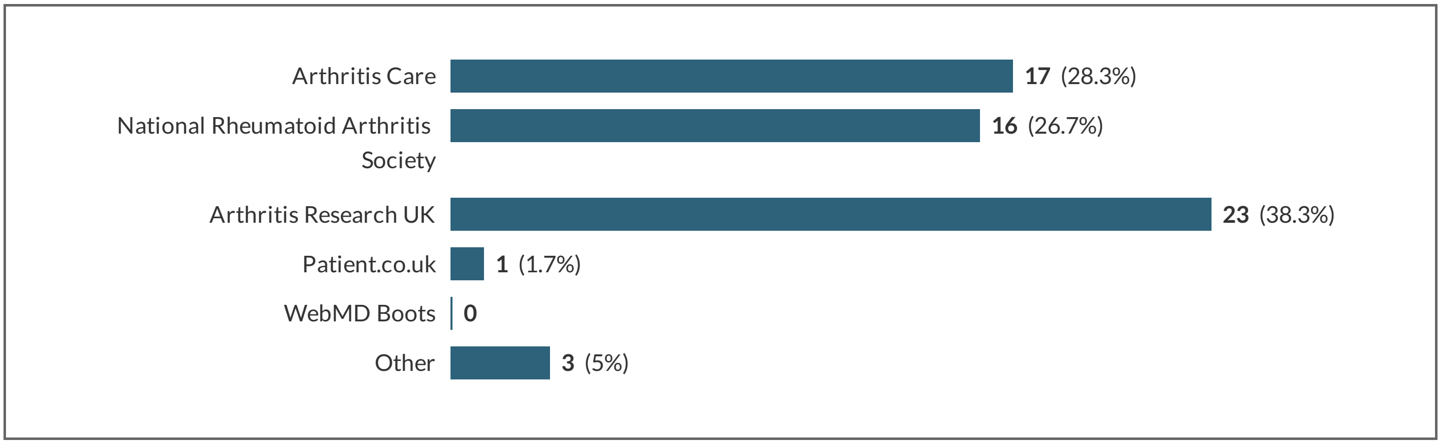

11.a If you selected Other, please specify:

Showing all 3 responses	
Shoemed in Stratford.	120812-120806-6760693
Footwear related websites	120812-120806-6760700
make sure www sites use are pref "UK" and are 'mainstream'.	120812-120806-6760710

12 From the options given,to what extent do you think they are effective.

12.1 Verbal Information

12.1.a Verbal Information - Please select from Very Effective to Don't KNow

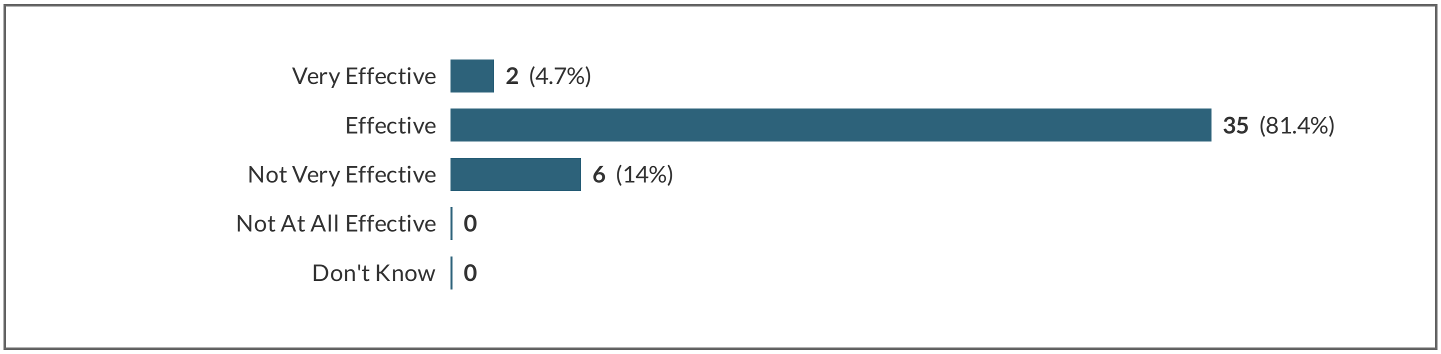

12.2 Written Information

12.2.a Written Information - Please select from Very Effective to Don't KNow

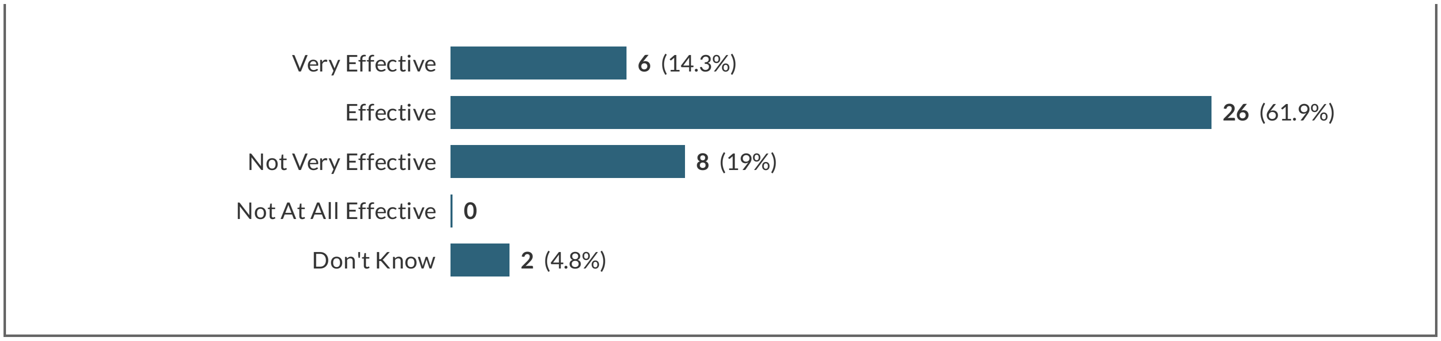

12.3 Group Education sessions

12.3.a Group Education sessions - Please select from Very Effective to Don't KNow

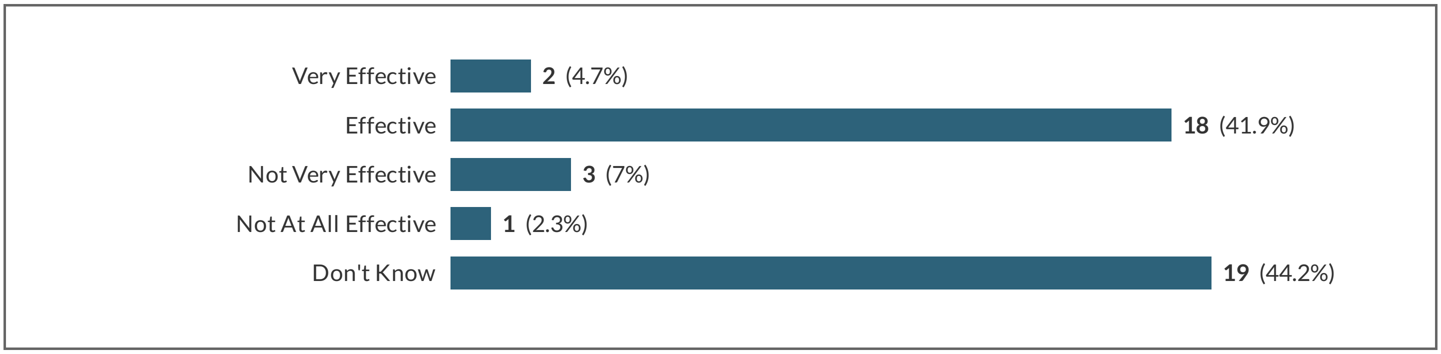

12.4 Audio-visual aids such as videos/demonstrations

12.4.a Audio-visual aids such as videos/demonstrations - Please select from Very Effective to Don't KNow

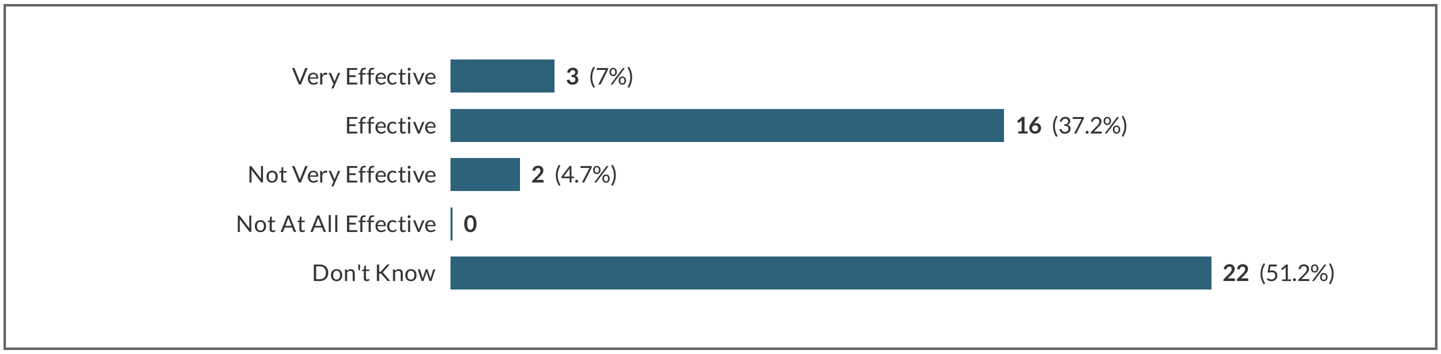

12.5 Web sites

12.5.a Web sites - Please select from Very Effective to Don't KNow

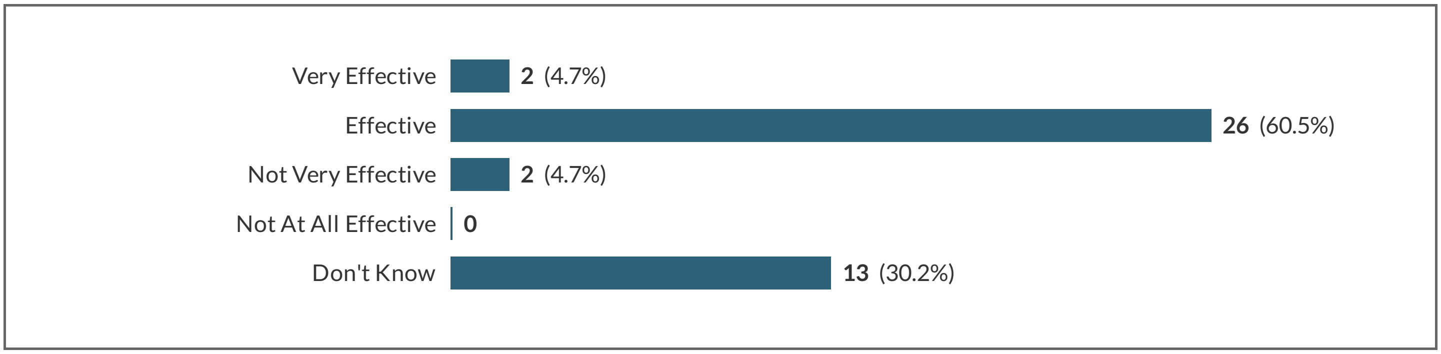

12.6 Combination of resources

12.6.a Combination of resources - Please select from Very Effective to Don't KNow

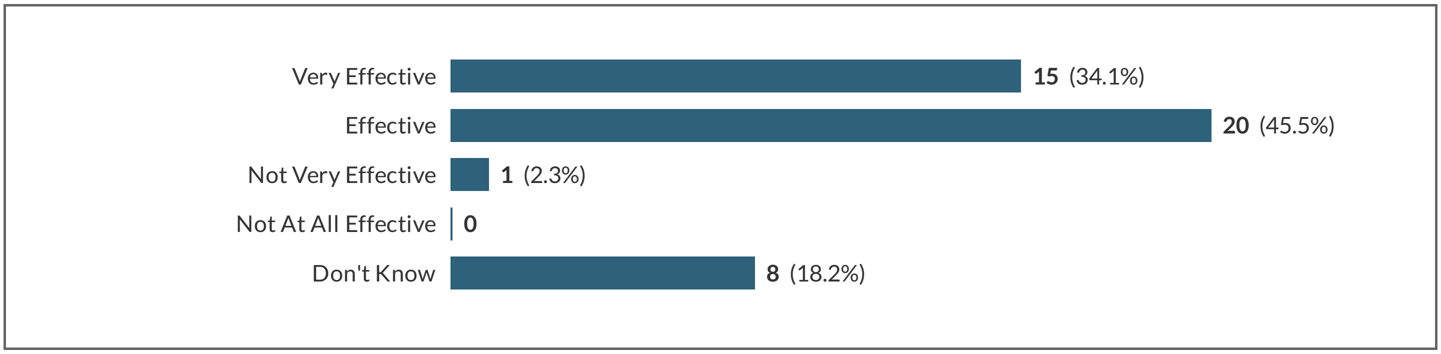

12.7 Other (in relation to additional methods you gave)

12.7.a Other (in relation to additional methods you gave) - Please select from Very Effective to Don't KNow

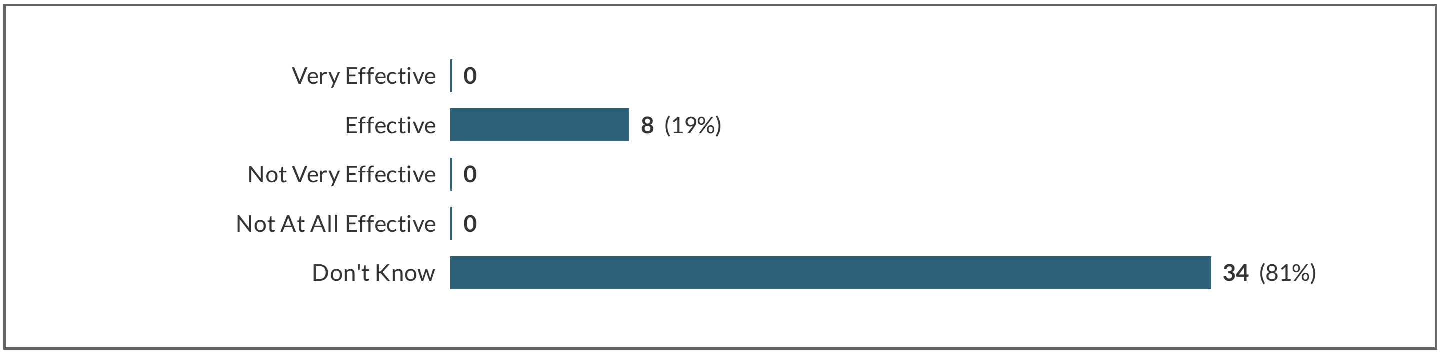

Section 4: The content of foot health education

13 To what extent do you think it is important for people with RA to know about the following areas of foot health education? Select from Very Important to Not Important for each item. They are in no particular order.

13.1 The role of the podiatrist in managing foot health

13.1.a The role of the podiatrist in managing foot health - Please select from Very Important to Not Important

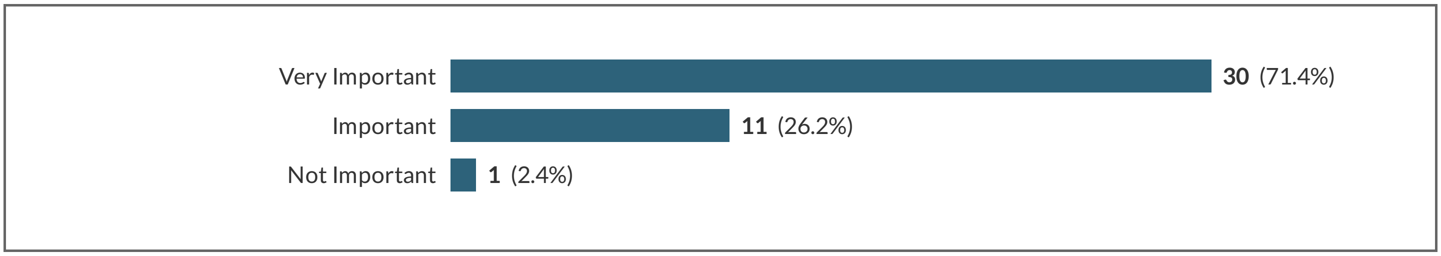

13.2 General disease related information (e.g what is RA? Causes etc..)

13.2.a General disease related information (e.g what is RA? Causes etc..) - Please select from Very Important to Not Important

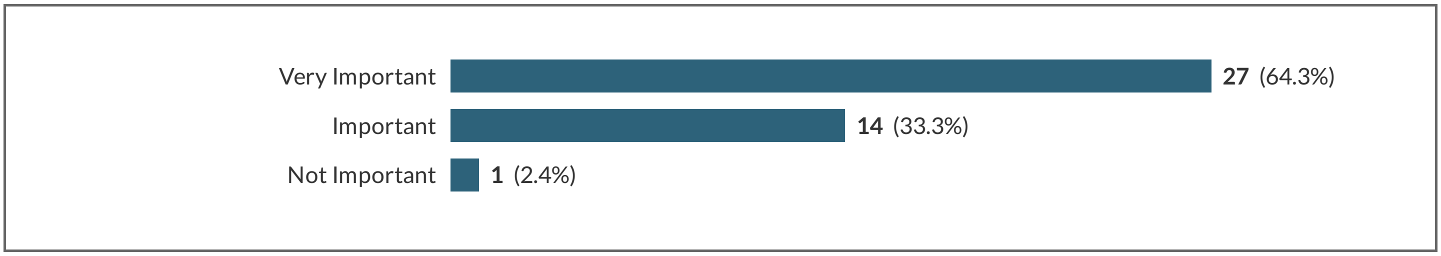

13.3 Information relating to the medication taken for RA and how it can affect the feet

13.3.a Information relating to the medication taken for RA and how it can affect the feet - Please select from Very Important to Not Important

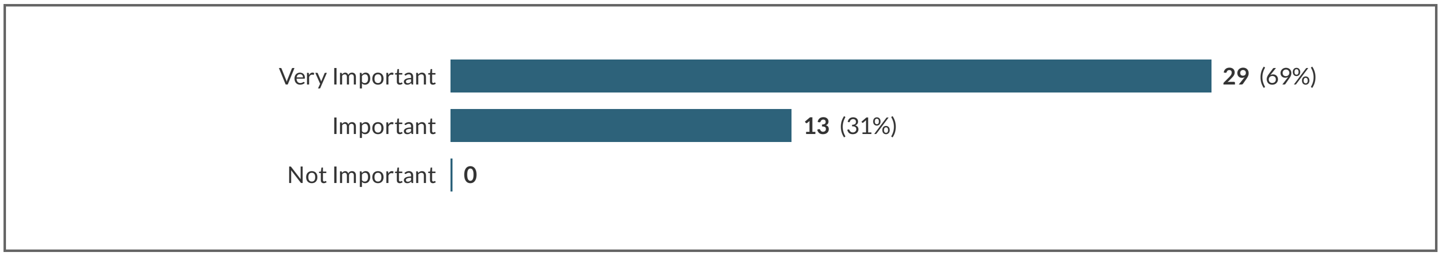

13.4 Signs and symptoms of foot problems related to RA

13.4.a Signs and symptoms of foot problems related to RA - Please select from Very Important to Not Important

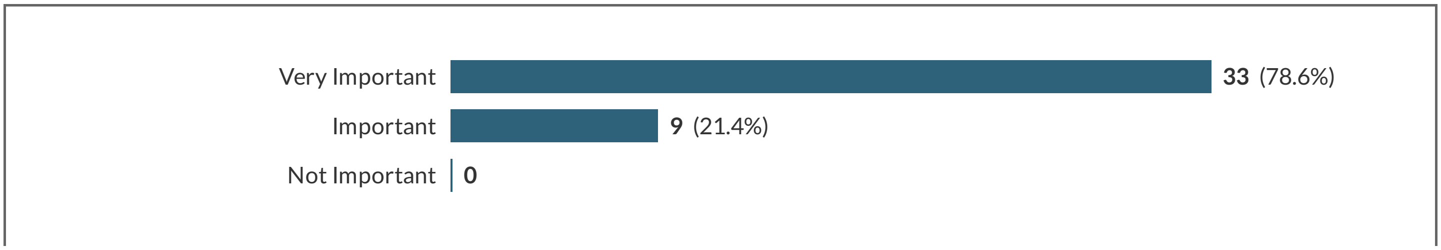

13.5 Contact details for podiatry services (when its an emergency, how and who to contact)

13.5.a Contact details for podiatry services (when its an emergency, how and who to contact) - Please select from Very Important to Not Important

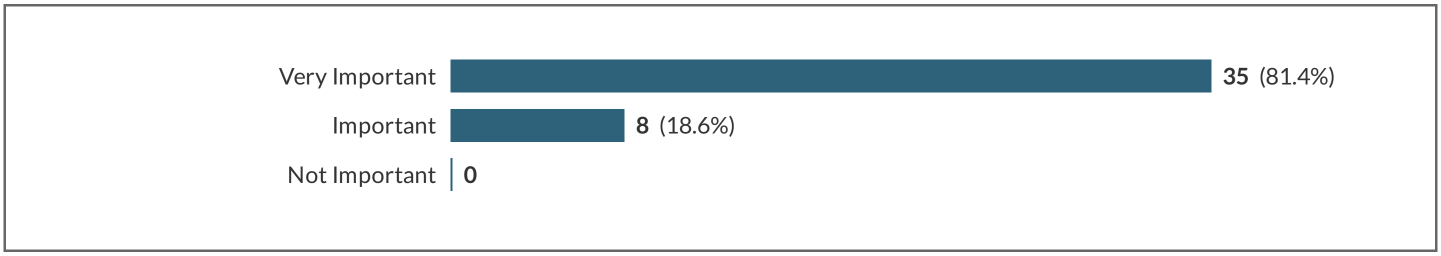

13.6 Management options relating to foot health

13.6.a Management options relating to foot health - Please select from Very Important to Not Important

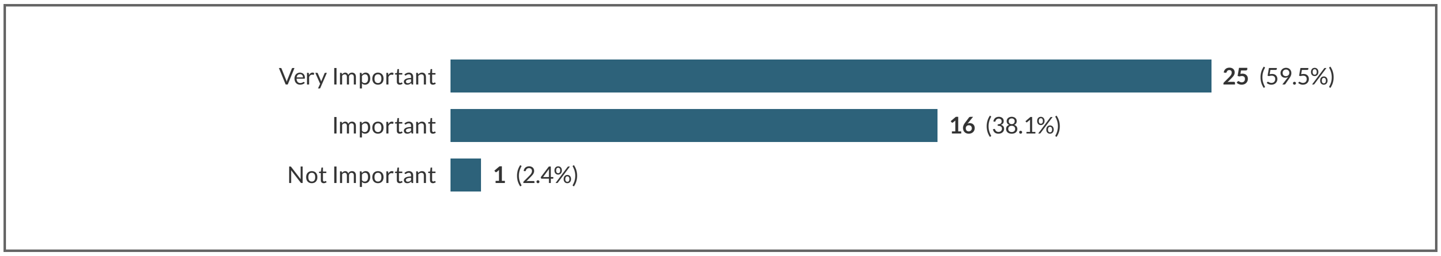

13.7 How to manage their own foot health (footwear advice, safe nail cutting practice, use of moisturiser..)

13.7.a How to manage their own foot health (footwear advice, safe nail cutting practice, use of moisturiser..) - Please select from Very Important to Not Important

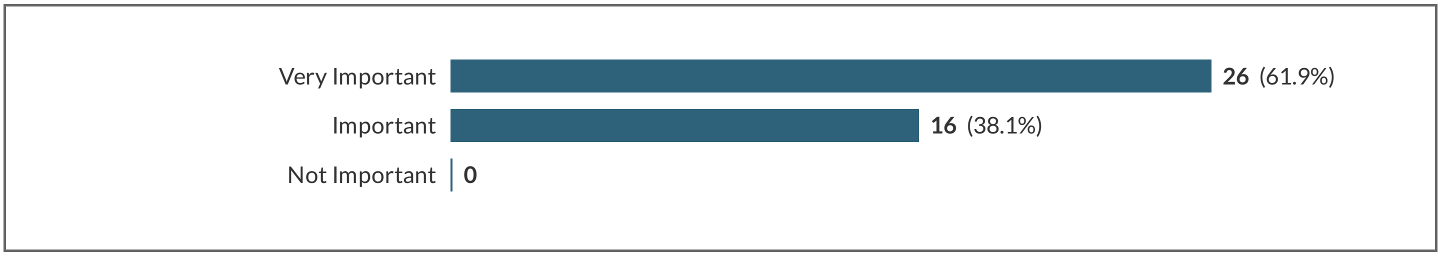

13.8 The consequences of not looking after their feet

13.8.a The consequences of not looking after their feet - Please select from Very Important to Not Important

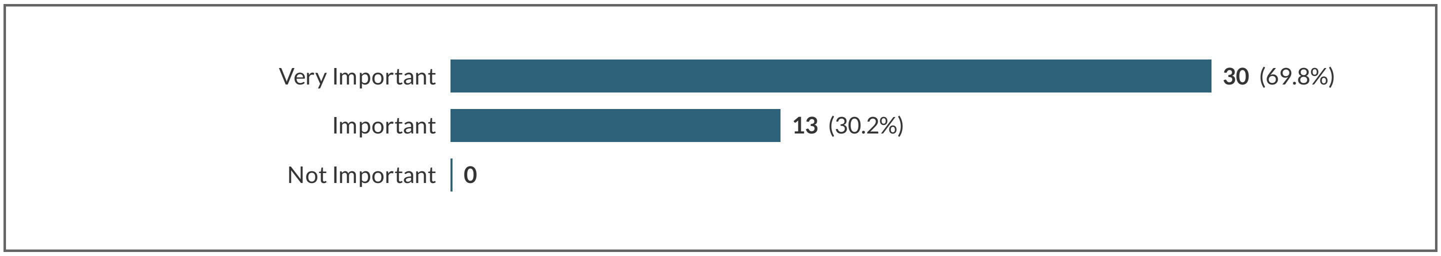

13.9 The role of other professions in managing foot health

13.9.a The role of other professions in managing foot health - Please select from Very Important to Not Important

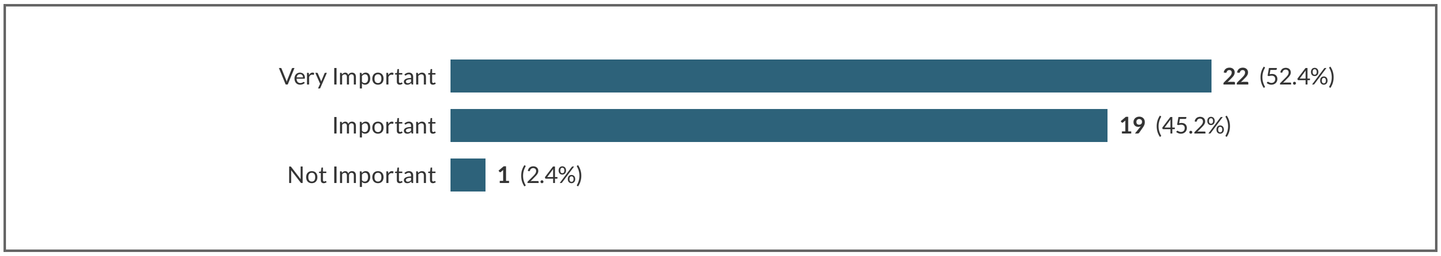

13.10 Information relating to Patient support groups/resources such as websites (e.g NRAS, Arthritis Care)

13.10.a Information relating to Patient support groups/resources such as websites (e.g NRAS, Arthritis Care) - Please select from Very Important to Not Important

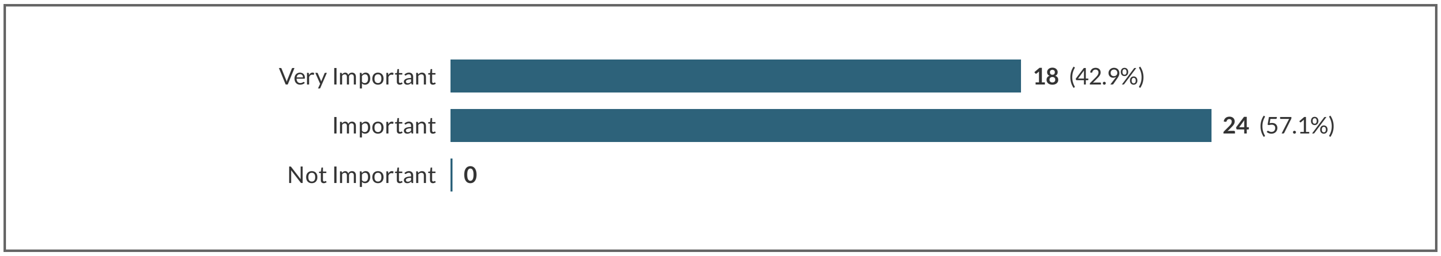

Section 5: The timing of foot health education

14 To what extent do you agree with the following statements in relation to the timing of foot health education provision.

14.1 Foot health education should be provided at the point of diagnosis of RA

14.1.a Foot health education should be provided at the point of diagnosis of RA - To what extent do you agree with the following statements

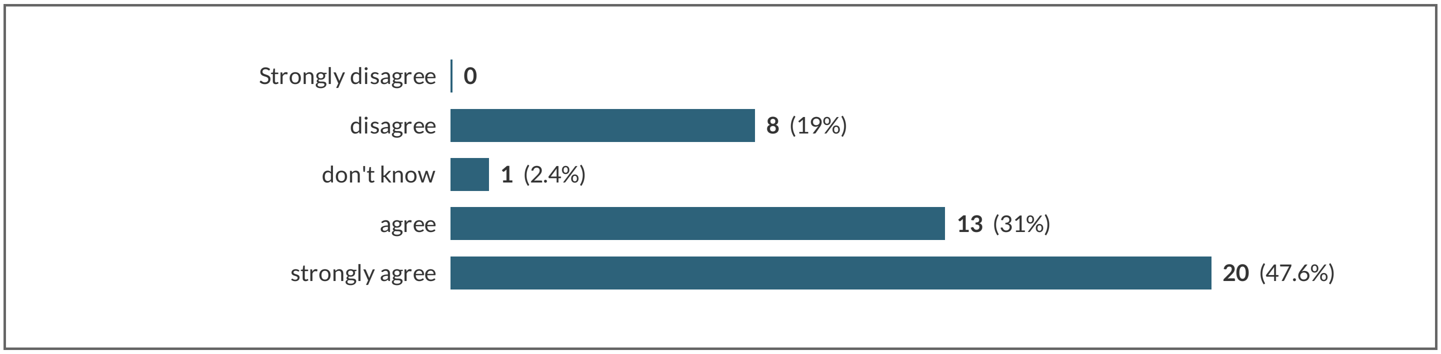

14.2 Foot health education should be provided only when you are asked for it

14.2.a Foot health education should be provided only when you are asked for it - To what extent do you agree with the following statements

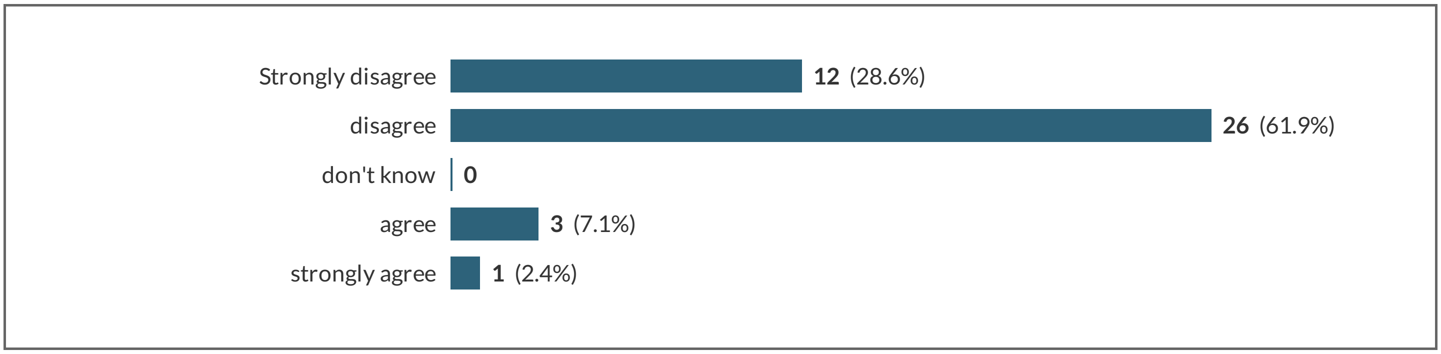

14.3 Foot health education should be provided when/if the person develops foot related symptoms

14.3.a Foot health education should be provided when/if the person develops foot related symptoms - To what extent do you agree with the following statements

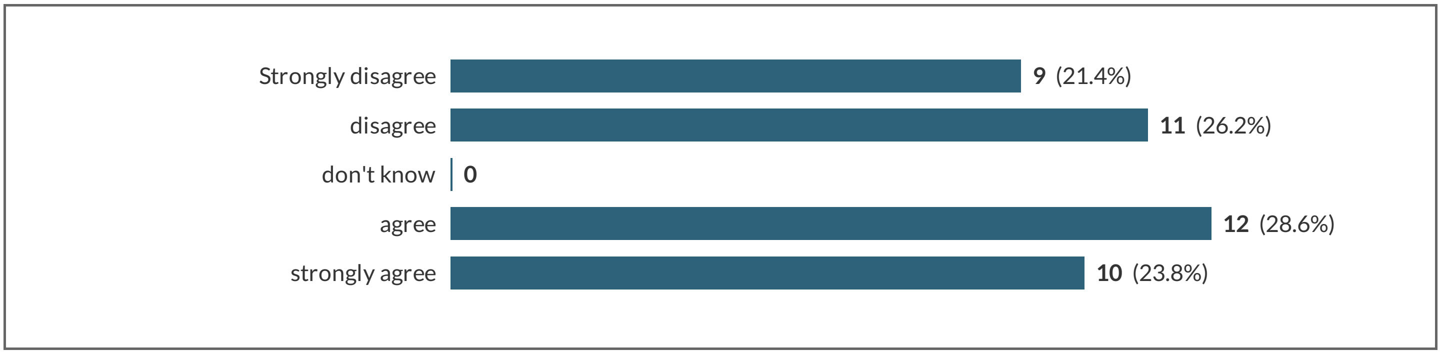

14.4 Foot health education should be provided at every available opportunity

14.4.a Foot health education should be provided at every available opportunity - To what extent do you agree with the following statements

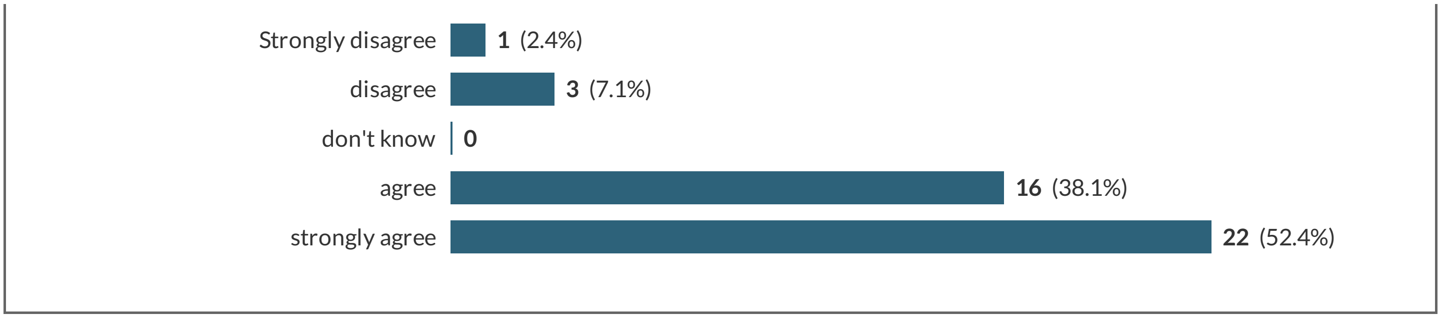

Section 6: Barriers to providing foot health education

15 To what extent do you agree with the following statements? Select one response for each item.

15.1 There is enough time during consultations to provide foot health education

15.1.a There is enough time during consultations to provide foot health education - Please select from Strongly Agree to Strongly Disagree for each item. If you are unsure please select 'Don't Know'. There is space in the final column for each item if you wish to add any other comment.

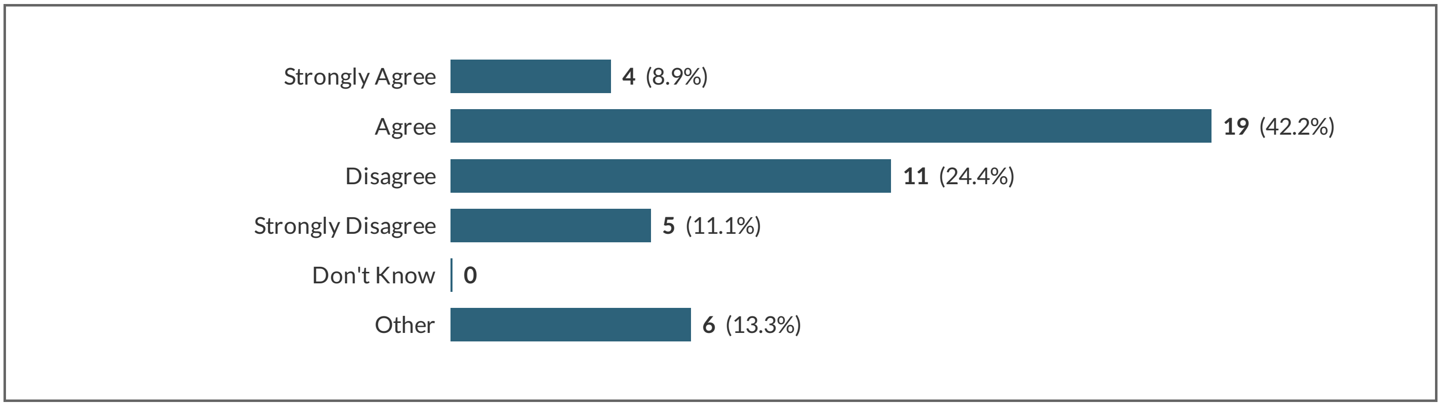

15.1.b There is enough time during consultations to provide foot health education - If you selected Other, please specify:

Showing 5 of 6 responses	
there can be enough time for small, focused, discussions on a particular area of health edcaution. there is not enough time to engage with the patient and determine THEIR individual fears,especations	120812-120806-6760689
talk while your working	120812-120806-6760696
This depends on how services have been set up. I am lucky enough to have a set up that allows time to explain, educate and negotiate. I would say however that podiatry services have very little time and resource for foot health education	120812-120806-6760697
We are always under pressure for time but foot health education should be made a priority.	120812-120806-6760707
caseloads, overbooking lateness, extras put enormous pressure on clinical appointments	120812-120806-6760711

15.2 You have access to RA-specific foot health information such as leaflets, provided either by your Trust or from Patient support organisations such as NRAS or Arthritis Research UK

15.2.a You have access to RA-specific foot health information such as leaflets, provided either by your Trust or from Patient support organisations such as NRAS or Arthritis Research UK - Please select from Strongly Agree to Strongly Disagree for each item. If you are unsure please select 'Don't Know'. There is space in the final column for each item if you wish to add any other comment.

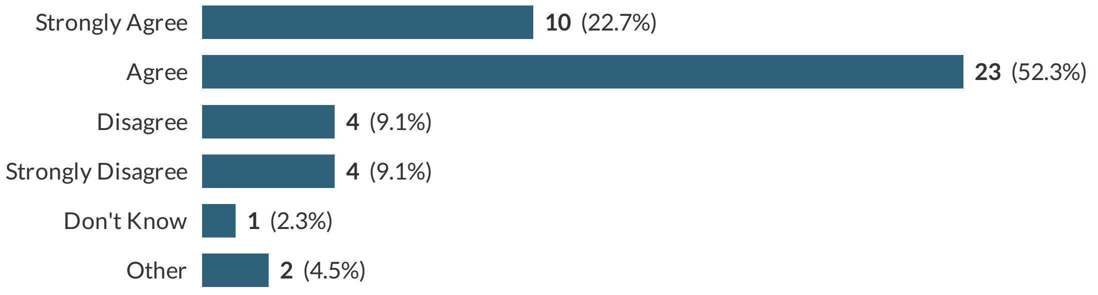

15.2.b You have access to RA-specific foot health information such as leaflets, provided either by your Trust or from Patient support organisations such as NRAS or Arthritis Research UK - If you selected Other, please specify:

Showing all 2 responses	
I have a limited supply from NRAS and the trust does not provide health leaflets.	120812-120806-6760707
Trust ones currently being developed. just provide ARC leaflets at moment	120812-120806-6760716

15.3 You are aware of any Group Education programmes that you could refer your patients into

15.3.a You are aware of any Group Education programmes that you could refer your patients into - Please select from Strongly Agree to Strongly Disagree for each item. If you are unsure please select 'Don't Know'. There is space in the final column for each item if you wish to add any other comment.

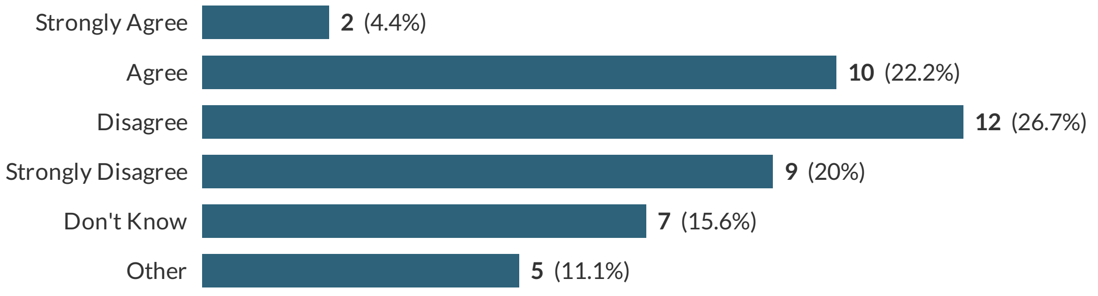

15.3.b You are aware of any Group Education programmes that you could refer your patients into - If you selected Other, please specify:

Showing all 5 responses	
not for RA in particula- we have 'pain managment, long term conditions' sessions	120812-120806-6760689
Decision was made to discontinue group sessions although do use fibromyalgia group sessions for those RA who have a fibromyalgic component in their disease	120812-120806-6760694
Arthritis care Groups - some don't do groups but they work if they do them.	120812-120806-6760696
Although not specific to Rheumatoid Arthritis	120812-120806-6760707
only for those with consultant in area not for those will cross boundary care	120812-120806-6760716

15.4 You have enough knowledge about how RA effects the feet in order to provide effective foot health education

15.4.a You have enough knowledge about how RA effects the feet in order to provide effective foot health education - Please select from Strongly Agree to Strongly Disagree for each item. If you are unsure please select 'Don't Know'. There is space in the final column for each item if you wish to add any other comment.

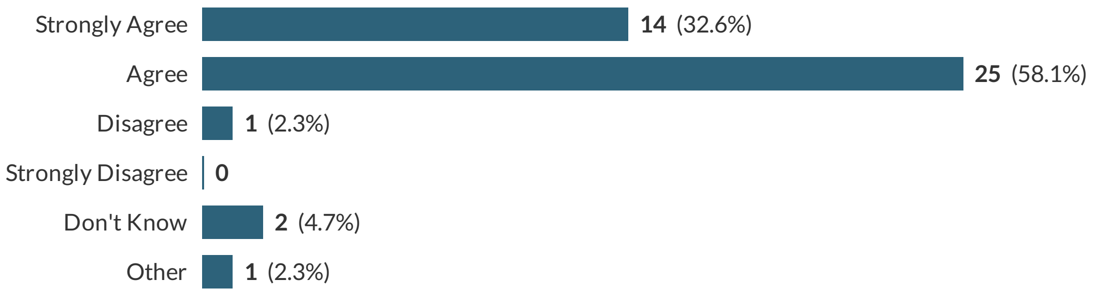

15.4.b You have enough knowledge about how RA effects the feet in order to provide effective foot health education - If you selected Other, please specify:

Showing 1 response	
RA can be very distruptive to the foot joints and I am unsure as to which extend suitable footwear can prevent problems. When problems do occur, RA patients in particular, are very good at finding ways to relief their symptroms. They are more receptive at that stage, I think.	120812-120806-6760698

15.5 The people that you manage with RA use the foot health education that you provide

15.5.a The people that you manage with RA use the foot health education that you provide - Please select from Strongly Agree to Strongly Disagree for each item. If you are unsure please select 'Don't Know'. There is space in the final column for each item if you wish to add any other comment.

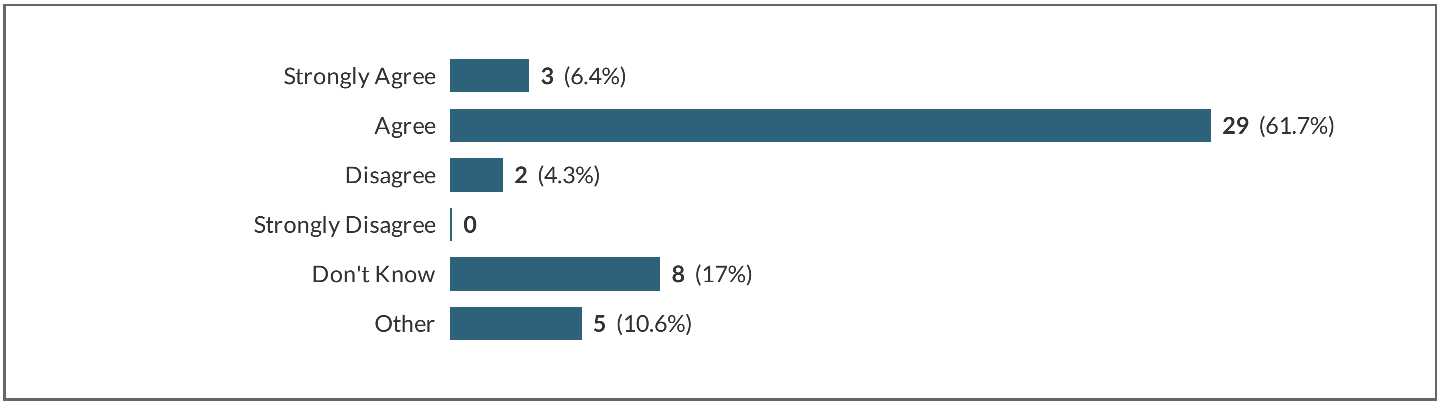

15.5.b The people that you manage with RA use the foot health education that you provide - If you selected Other, please specify:

Showing all 5 responses	
other than some female patients and footwear advice	120812-120806-6760694
Over time, not straight away.	120812-120806-6760698
Some do, some don't	120812-120806-6760700
Sometimes.	120812-120806-6760707
in the main yes but they have gross foot deformity due to RA for many years and sometimes object to styles of shoes available	120812-120806-6760716

16 If people with RA DO NOT use the foot health education that you provide, is this because (please select all that apply)...

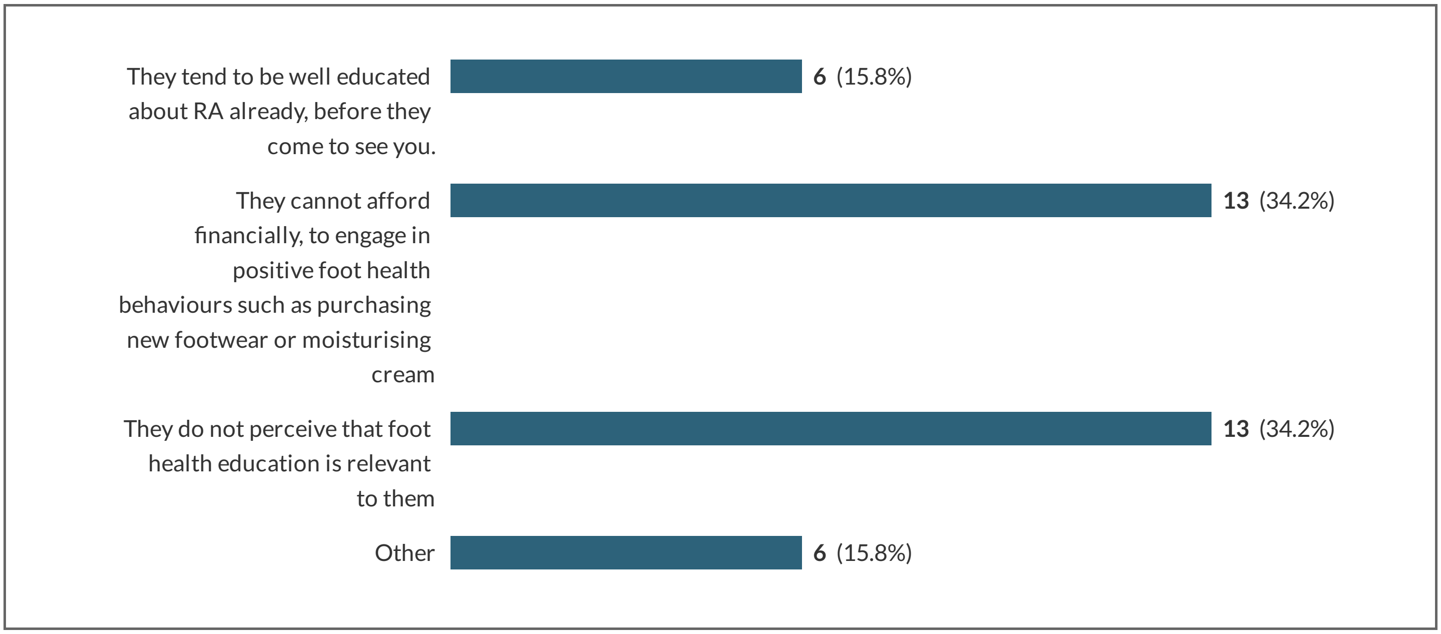

16.a If you selected Other, please specify:

Showing 5 of 6 responses	
they are unable to due to physical constraints and lack of help by others	120812-120806-6760695
some do some don't - barriers are cost, motivation - it won't happen to me or I know that but it's too late or just too much bother. Until it happens.	120812-120806-6760696
People find it generally difficult to alter behaviours and this includes changing footwear type. This is more of a proble with women rather than with men. Additionally, elderly people can find that social barriers hinders them from wearing trainer-type shoes but don't want to wear 'shoes for old people'either.	120812-120806-6760698
Often receptiveness to HE relates to symptom only. People experiencing foot pain can appreciate the relevance and use of FHE. Often the newly diagnosed without foot pain can't see the relevance to them and disregard it. FHE is best given at all opportunities but I think best received when the person given FHE has a personal interest	120812-120806-6760705
Excuses I hear most often:<br>They are young and do not want to wear certain shoe types.<br>Their occupation dictates a shoe type.<br>Only flip flops are comfortable.<br>'They might not be good for me but they're comfortable!'	120812-120806-6760707

17 This free text box is for you to add any additional comments or information that you feel is relevant and has not been addressed by this survey. Thank you.

Showing 5 of 13 responses	
Section 2 Q8 titles appear to have a typo as both ends of the scale are strongly disagree. I meant strongly AGREE for my response.<br>Good luck,	120812-120806-6760685
Re timing, foot health education is important in early diagnosis but patients are often overwhelmed with new information at this time and therefore foot health education is perhaps best delivered as part of a staggered education approach by the team.	120812-120806-6760686
Section number 2 has choices wrong both strongly disagree	120812-120806-6760687
A barrier to the importence of foot health education is the foot involvement in RA is not given the same priority (by the MDT) as other aspects of care.	120812-120806-6760691
Working in the private sector I have to refer them into the NHS anyway, it would be immoral not to.	120812-120806-6760696
