# Supplementary material for: Foot health education provision for people with rheumatoid arthritis–an online survey of UK podiatrists’ perceptions
Source: J Foot Ankle Res. 2016 Apr 26;9:13. doi: 10.1186/s13047-016-0145-6 (PMC4845353; doi:10.1186/s13047-016-0145-6)
Supplement: Additional file 2: — Table of results from statistical analyses. The table illustrates the influence of the years qualified, age range and gender of the podiatrists on their survey responses. (DOCX 187 kb) [file 13047_2016_145_MOESM2_ESM.docx]

Appendix 2: Statistical analyses: influence of Podiatrists’ Years Qualified, Age Range and Gender on survey responses.

(* denotes statistical significance of *p=*<0.05)

| **Section 1: the aim of foot health education is to:** | Years qualified | **Fishers Exact Test Value** | **Exact Sig. (2-sided)** | Age Range | **Fishers Exact Test Value** | **Exact Sig. (2-sided)** | Gender | **Fishers Exact Test Value** | **Exact Sig. (2-sided)** |
| --- | --- | --- | --- | --- | --- | --- | --- | --- | --- |
| Allow informed consent | Years Qualified range | 15.943 | 0.472 | Age Range | 11.873 | 0.59 | Gender | 7.138 | **0.047*** |
| Facilitate informed choices about treatment options |  | 23.856 | 0.062 |  | 14.822 | 0.536 |  | 11.463 | **0.005*** |
| Enable them to manage their own foot health |  | 6.001 | 0.339 |  | 3.363 | 0.579 |  |  | **0.013*** |
| Educate them about how RA can affect their feet |  | 9.329 | **0.051*** |  | 2.69 | 0.714 |  |  | **0.033*** |
| Inform about information resources they can access |  | 13.551 | 0.24 |  | 8.218 | 0.742 |  | 4.258 | 0.097 |
| **Section 2: Methods of FHE provision** |  |  |  |  |  |  |  |  |  |
| Do you provide FHE to people with RA |  | 6.079 | 1 |  | 5.386 | 0.595 |  |  | 0.262 |
| Do you provide verbal information |  | 3.647 | 1 |  | 3.086 | 1 |  |  | 0.064 |
| Do you provide Written information |  | 2.79 | 0.81 |  | 3.435 | 0.507 |  |  | 0.713 |
| Do you provide Group Education |  | 4.512 | 0.472 |  | 2.916 | 0.615 |  |  | 0.644 |
| Do you use Audio-visual aids such as demonstrations/videos |  | 5.194 | 0.619 |  | 3.086 | 1 |  |  | 0.46 |
| Do you direct patients to Arthritis related websites |  | 6.33 | 0.229 |  | 2.114 | 0.878 |  |  | 0.483 |
| If you use websites do you use: Arthritis care |  | 5.709 | 0.305 |  | 6.221 | 0.131 |  |  | 1 |
| If you use websites do you use: NRAS |  | 8.648 | 0.073 |  | 2.238 | 0.79 |  |  | 0.485 |
| If you use websites do you use: Arthritis Research UK |  | 7.315 | 0.15 |  | 2.535 | 0.771 |  |  | 0.504 |
| If you use websites do you use: Patient.co.uk |  | 6.079 | 1 |  | 4.997 | 1 |  |  | 1 |
| If you use websites do you use: WebMD Boots |  | no responses | no responses |  | no Reponses | no responses |  | no responses | no responses |
| If you use websites do you use: other |  | 6.116 | 0.31 |  | 6.746 | 0.12 |  |  | **0.014*** |
| How effective do you think verbal information is |  | 14.668 | 0.081 |  | 11.781 | 0.131 |  | 5.28 | 0.069 |
| How effective do you think written information is |  | 14.662 | 0.513 |  | 11.898 | 0.532 |  | 2.912 | 0.413 |
| How effective do you think group education is |  | 26.807 | 0.114 |  | 17.337 | 0.629 |  | 2.176 | 0.826 |
| How effective do you think audio-visual aids are |  | 12.094 | 0.88 |  | 9.572 | 0.881 |  | 1.349 | 0.929 |
| How effective do you think websites are |  | 13.302 | 0.825 |  | 10.863 | 0.784 |  | 4.355 | 0.228 |
| How effective do you think a combination of resources are |  | 16.295 | 0.5 |  | 11.729 | 0.699 |  | 3.041 | 0.414 |
| **Section 3: How important is it for people with RA to know about:** |  |  |  |  |  |  |  |  |  |
| The role of the podiatrist in managing their foot health |  | 13.379 | 0.25 |  | 7.943 | 0.74 |  | 3.045 | 0.203 |
| General disease related information |  | 8.026 | 0.99 |  | 7.756 | 0.804 |  | 0.588 | 1 |
| How RA-related medication can affect the feet |  | 4.269 | 0.552 |  | 2.624 | 0.737 |  |  | 0.713 |
| The signs and symptoms of foot problems related to RA |  | 1.922 | 0.972 |  | 3.596 | 0.496 |  |  | **0.038*** |
| Contact details for podiatry services |  | 1.88 | 0.961 |  | 1.528 | 0.927 |  |  | **0.063** |
| Management options relating to foot health |  | 8.138 | 0.984 |  | 9.677 | 0.435 |  | 11.379 | **0.003*** |
| How to manage their own foot health |  | 5.238 | 0.387 |  | 3.803 | 0.45 |  |  | **0.07*** |
| The consequences of not looking after their feet |  | 2.909 | 0.797 |  | 3.333 | 0.558 |  |  | 0.243 |
| The role of other health professions in managing foot health |  | 9.436 | 0.874 |  | 11.148 | 0.228 |  | 0.506 | 1 |
| Information relating to patient support groups/websites |  | 6.321 | 0.235 |  | 3.594 | 0.481 |  |  | 0.299 |
| **Section 4: Timing - FHE should be provided:** |  |  |  |  |  |  |  |  |  |
| At the point of diagnosis |  | 14.723 | 0.707 |  | 11.496 | 0.738 |  | 1.975 | 0.618 |
| Only when you are asked for it |  | 23.408 | **0.034*** |  | 14.954 | 0.317 |  | 1.357 | 0.899 |
| When or if the person develops foot-related symptoms |  | 21.809 | **0.022*** |  | 13.141 | 0.257 |  | 0.385 | 1 |
| At every available opportunity |  | 16.438 | 0.54 |  | 15.541 | 0.248 |  | 2.979 | 0.476 |
| **Section 5: Barriers to FHE provision** |  |  |  |  |  |  |  |  |  |
| There is enough time during consultations to provide FHE |  | 20.078 | 0.408 |  | 14.364 | 0.687 |  | 1.833 | 0.842 |
| You have access to RA specific foot health information such as leaflets, provided by your Trust or patient support organisations |  | 18.916 | 0.78 |  | 18.871 | 0.332 |  | 8.74 | **0.031*** |
| You are aware of any Group Education programmes that you could refer your patients into |  | 23.621 | 0.598 |  | 25.045 | 0.094 |  | 4.885 | 0.409 |
| You have enough knowledge about how RA affects the feet to provide effective FHE |  | 19.134 | 0.275 |  | 13.518 | 0.556 |  | 3.993 | 0.259 |
| The people that you manage with RA use the FHE that you provide |  | 12.947 | 0.785 |  | 12.358 | 0.52 |  | 3.434 | 0.278 |
| People don't use your FHE because they are already well educated |  | 2.818 | 0.801 |  | 5.273 | 0.237 |  |  | 1 |
| People don't use your FHE because they cannot afford to for financial reasons |  | 6.304 | 0.228 |  | 1.947 | 0.875 |  |  | 0.464 |
| People don't use your FHE because they do not perceive that it is relevant to them |  | 5.687 | 0.302 |  | 1.524 | 1 |  |  | 0.713 |
| People don't use your FHE because of other reasons |  | 7.523 | 0.128 |  | 2.849 | 0.659 |  |  | 0.303 |
