# Supplementary material for: Foot health education provision for people with rheumatoid arthritis–an online survey of UK podiatrists’ perceptions
Source: J Foot Ankle Res. 2016 Apr 26;9:13. doi: 10.1186/s13047-016-0145-6 (PMC4845353; doi:10.1186/s13047-016-0145-6)
Supplement: Additional file 3: — Free text comments from RA FHE survey of podiatrists. This file shows the free text comments taken from Rheumatoid Arthritis foot health education survey for practitioners. (DOCX 24 kb) [file 13047_2016_145_MOESM3_ESM.docx]

**Free text comments taken from Rheumatoid Arthritis foot health education survey for practitioners.**

**15.1.b –There is enough time during consultations to provide foot health education – If you selected Other, please specify:**

| **Showing all 6 responses** |
| --- |
| 1. there can be enough time for small, focused, discussions on a particular area of health edcaution. there is not enough time to engage with the patient and determine THEIR individual fears,especations |
| 1. talk while your working |
| 1. This depends on how services have been set up. I am lucky enough to have a set up that allows time to explain, educate and negotiate. I would say however that podiatry services have very little time and resource for foot health education |
| 1. We are always under pressure for time but foot health education should be made a priority. |
| 1. caseloads, overbooking lateness, extras put enormous pressure on clinical appointments |
| 1. pertinent to the presenting problem or request from the patient in reality is what health ed is given during a consultation |

**15.2.b -You have access to RA-specific foot health information such as leaflets, provided either by your Trust or from Patient support organisations such as NRAS or Arthritis Research UK - If you selected Other, please specify:**

| **Showing all 2 responses** |
| --- |
| 1. I have a limited supply from NRAS and the trust does not provide health leaflets. |
| 1. Trust ones currently being developed. just provide ARC leaflets at moment |

**15.3.b -You are aware of any Group Education programmes that you could refer your patients into - If you selected Other, please specify:**

| **Showing all 5 responses** |
| --- |
| 1. not for RA in particula- we have 'pain managment, long term conditions' sessions |
| 1. Decision was made to discontinue group sessions although do use fibromyalgia group sessions for those RA who have a fibromyalgic component in their disease |
| 1. Arthritis care Groups - some don't do groups but they work if they do them. |
| 1. Although not specific to Rheumatoid Arthritis |
| 1. only for those with consultant in area not for those will cross boundary care |

**15.4.b - You have enough knowledge about how RA effects the feet in order to provide effective foot health education - If you selected Other, please specify:**

| **Showing 1 response** | |
| --- | --- |
| 1. RA can be very distruptive to the foot joints and I am unsure as to which extend suitable footwear can prevent problems. When problems do occur, RA patients in particular, are very good at finding ways to relief their symproms. They are more receptive at that stage, I think. |  |

### 16. The people that you manage with RA use the foot health education that you provide - If you selected Other, please specify:

| **Showing all 5 responses** |
| --- |
| 1. other than some female patients and footwear advice |
| 1. Over time, not straight away. |
| 1. Some do, some don't |
| 1. Sometimes. |
| 1. in the main yes but they have gross foot deformity due to RA for many years and sometimes object to styles of shoes available |

### Question 16: If people with RA DO NOT use the foot health education that you provide, is this because (please select all that apply)...

### Question 16.a: If you selected Other, please specify:

| **Showing all 6 responses** |
| --- |
| 1. they are unable to due to physical constraints and lack of help by others |
| 1. some do some don't - barriers are cost, motivation - it won't happen to me or I know that but it's too late or just too much bother. Until it happens. |
| 1. People find it generally difficult to alter behaviours and this includes changing footwear type. This is more of a proble with women rather than with men. Additionally, elderly people can find that social barriers hinders them from wearing trainer-type shoes but don't want to wear 'shoes for old people'either. |
| 1. Often receptiveness to HE relates to symptom only. People experiencing foot pain can appreciate the relevance and use of FHE. Often the newly diagnosed without foot pain can't see the relevance to them and disregard it. FHE is best given at all opportunities but I think best received when the person given FHE has a personal interest |
| 1. Excuses I hear most often: They are young and do not want to wear certain shoe types.  Their occupation dictates a shoe type. Only flip flops are comfortable. 'They might not be good for me but they're comfortable!' |
| 1. usually footwear related problems and would rather wear their own instead of hospital footwear |

### Question 17: This free text box is for you to add any additional comments or information that you feel is relevant and has not been addressed by this survey. Thank you.

|  |
| --- |
| 1. Re timing, foot health education is important in early diagnosis but patients are often overwhelmed with new information at this time and therefore foot health education is perhaps best delivered as part of a staggered education approach by the team. |
| 1. A barrier to the importence of foot health education is the foot involvement in RA is not given the same priority (by the MDT) as other aspects of care. |
| 1. Working in the private sector I have to refer them into the NHS anyway, it would be immoral not to. |
| 1. FHE in RA is a long-term investment. Podiatry and NHS Trust managers only see the end of the current financial year. Perhaps a survey to pod managers to ask how much resource they allocate to the above might be an area to look at, as 50% of the positive responses in this survey have been achieved by my own dedication, commitment and unpaid work!!! |
| 1. I don't think that enough effort has been made to offer people with altered foot shape desirable custome-made footwear; neither is the current fashion accommodating the -so called- 'deformed feet'. |
| 1. I work in private practice. RA patients I see are already managed within the NHS. My role as I see it is to reiterate health care advice and refer to medical/nursing practitioners as required |
| 1. Patient education can be a challenge depending on age, duration of disease, previous foot surgery and level of pain etc. In my experience, Pain can be generally a good motivating factor for patients to listen to advice and education. |
| 1. foot heath foot ed can give "worse case" scenario, can sound like "threat". To give info to eg. JIA like that is very negative. Little bytes are better that big chunks!! |
| 1. Too foot focussed? No mention of the systemic impact of RA and for example cardiovascular and peripheral vascular risks. |
| 1. as podiatrists we perceive foot health education as a need at first diagnosis. If there are no evident visual or symptomatic foot problems at this stage it is difficult to convince patients/other professionals that podiatry intervention is important at this stage. in my experience patients often attend podiatry 'once the damage is done' |
| 1. Types of education and those who respond to it caries from person to person on the whole I find this group more receptive re thier disease than for examples patients with diabetes. |
